# Toxin-Antitoxin Systems: A Tool for Taxonomic Analysis of Human Intestinal Microbiota

**DOI:** 10.3390/toxins12060388

**Published:** 2020-06-12

**Authors:** Ksenia M. Klimina, Viktoriya N. Voroshilova, Elena U. Poluektova, Vladimir A. Veselovsky, Roman A. Yunes, Aleksey S. Kovtun, Anna V. Kudryavtseva, Artem S. Kasianov, Valery N. Danilenko

**Affiliations:** 1 Vavilov Institute of General Genetics Russian Academy of Sciences, 119991 Moscow, Russia; vorosviktoriya@yandex.ru (V.N.V.); epolu@vigg.ru (E.U.P.); romanyunes@gmail.com (R.A.Y.); kovtunas25@gmail.com (A.S.K.); artem.kasianov@gmail.com (A.S.K.); valerid@vigg.ru (V.N.D.); 2 Federal Research and Clinical Center of Physical-Chemical Medicine of Federal Medical Biological Agency, 119435 Moscow, Russia; djdf26@gmail.com; 3 Moscow Institute of Physics and Technology, Dolgoprudny, 141701 Moscow, Russia; 4 Engelhardt Institute of Molecular Biology, Russian Academy of Sciences, 119991 Moscow, Russia; rhizamoeba@mail.ru; 5 Faculty of Ecology, International Institute for Strategic Development of Sectoral Economics, Peoples’ Friendship University of Russia (RUDN University), 117198 Moscow, Russia

**Keywords:** toxin-antitoxin systems, metagenomes, gut microbiota, strain identification

## Abstract

The human gastrointestinal microbiota (HGM) is known for its rich diversity of bacterial species and strains. Yet many studies stop at characterizing the HGM at the family level. This is mainly due to lack of adequate methods for a high-resolution profiling of the HGM. One way to characterize the strain diversity of the HGM is to look for strain-specific functional markers. Here, we propose using type II toxin-antitoxin systems (TAS). To identify TAS systems in the HGM, we previously developed the software TAGMA. This software was designed to detect the TAS systems, MazEF and RelBE, in lactobacilli and bifidobacteria. In this study, we updated the gene catalog created previously and used it to test our software anew on 1346 strains of bacteria, which belonged to 489 species and 49 genera. We also sequenced the genomes of 20 fecal samples and analyzed the results with TAGMA. Although some differences were detected at the strain level, the results showed no particular difference in the bacterial species between our method and other classic analysis software. These results support the use of the updated catalog of genes encoding type II TAS as a useful tool for computer-assisted species and strain characterization of the HGM.

## 1. Introduction

The human microbiota (HM) refers to the multitude of commensal, symbiotic and pathogenic microorganisms inhabiting the human body. It consists of multiple ecosystems of microorganisms that coevolved to adapt to each other as well as to the hosts colonized by them. The gut microbiota (GM), which constitutes the largest and most significant part of the HM, plays an important role in the development and normal functioning of endocrine and nervous systems, which is carried out via its structural components and metabolites. The GM is also involved in nutrition, metabolism and defense against pathogens [1].

The HM consists of viruses, archaea, protozoa, fungi and bacteria, which is the largest and most studied group. The number of bacterial cells inhabiting the gastro-intestinal tract alone hovers around tens of trillions [2]. Today a staggering number of 2776 identified species has been associated with humans, which probably only accounts for less than half of the total number [3]. An estimated 500–1000 species of bacteria exist in the human body at any one time, and the number of strains could be orders of magnitude greater than this [1].

Microbiota alterations can be seen as an indicator of human health status. Indeed, many studies have found correlations between the GM composition and inflammatory bowel diseases, some forms of cancer, metabolic disorders including type I and type II diabetes and psychiatric and neurodegenerative diseases [4]. 105 different types of disorders have been associated with changes in the GM [5]. Therefore, the characterization of the taxonomic composition and functional profile of the GM remains an important practical task allowing the prevention and effective treatment of diseases.

Nowadays, the GM diversity can be characterized either by carrying out whole metagenome sequencing (WMS) or by amplifying the 16S rRNA genes using PCR. The approach of targeted sequencing of the 16S rRNA gene for taxonomic classification has its limitations since a small region of the 16S rRNA sequence of the gene is used, which gives resolution only to the genus [6]. High-throughput sequencing of the full 16S gene with sufficient accuracy to discriminate between copy variants has until recently been constrained by a lack of available sequencing technologies. The advent of long-read approaches on Nanopore and PacBio platforms has changed this. Johnson and co-authors demonstrate that appropriate handling of high-throughput, full-length 16S sequence data has the potential to enable accurate classification of individual organisms at very high taxonomic resolution [7]. The approach of WMS, due to its accuracy, is a powerful and advanced tool. Although WMS does not always allow us to restore the genome sequences of all bacterial strains constituting a microbiome, the use of additional biomarkers for taxonomic classification can render this task feasible. After carrying out WMS, a few taxonomic approaches exist: (1) methods based on analyzing sequence similarity, (2) biomarker-based methods, and (3) methods based on analyzing the parameters of the sequencing reads, such as the distribution of k-mer frequencies. The first method, which is based on the analysis of sequence similarity, compares the obtained reads with a reference sequence database. We can cite the software MegaBLAST [PMID:12776603], Megan [PMID:17255551] and PhymmBL [PMID:21527926] as useful examples of the principle mentioned above. As for the biomarker-based methods, only a set of genetic markers as opposed to whole genomic sequences is used as a reference. MetaPhlAn [PMID:22688413] is the most popular software with an embedded biomarker-based method of analysis of this kind. The third type of method is diverse since it includes many variations; however, the approach based on the analysis of the composition of k-mers in the reference sequences and the reads in a sample remains at the core. Kraken is one of the most challenging software of this type [PMID: 24580807]. We must note that the dominant trend of development favors speed over the level of taxonomic resolution. Unfortunately, at this point, the developed software does not allow going beyond the species-level characterization of samples.

Toxin-antitoxin systems (TAS) are genetic modules usually consisting of two genes encoding a stable toxin and labile antidote—antitoxin (protein or RNA). The toxin either kills the bacterial cells or leads to their reversible growth arrest; the mechanism of action of toxins vary, even though they are nucleases in most cases [8]. TAS are localized on plasmids, phages and chromosomes and are widespread in bacteria and archaea. However, the biological function of the majority of them is still elusive and contradictory. There is evidence that they are involved in post-segregational killing, abortive infection, bacterial persistence/antibiotic tolerance, biofilm formation, bacterial virulence and general stress response. Antitoxins are likely global regulators of cell processes. TAS are divided into six main types based on the nature of the antitoxin and the mechanisms of its inhibitory action [9]. In type II TAS both components—toxin and antitoxin—are proteins, and antitoxin directly suppresses the activity of the toxin. This class of TAS appears to be the most abundant in bacterial genomes [10]. Type II TAS are divided into families based on the similarity between the amino acid sequences of toxins and antitoxins [11,12]. However, hybrid systems, in which the toxin-antitoxin (TA)locus contains toxins and antitoxins from different families, have been discovered. Therefore, there is also a separate classification for the families of toxins and antitoxins [13].

Previously, we demonstrated that type II TAS of the superfamilies MazEF and RelBE are present in the genera *Bifidobacterium* and *Lactobacillus* and that different combinations of these systems, due to their high specificity, can be used for species-level and even strain-level identification [14,15,16,17]. We developed the software TAGMA (Toxin Antitoxin Genes for Metagenomes Analyses), based on a gene catalog of TAS MazEF and RelBE of lactobacilli and bifidobacteria, which was tested on 152 metagenomes and proven effective for species and strain identification [18]. The objective of this study was to expand the catalog of TA systems to encompass the main species inhabiting the gastrointestinal tract of humans and to validate the TAGMA software efficiency by carrying out taxonomic classification on the species and strain levels.

## 2. Results

### 2.1. Creating a Database

The database of TA systems that we built previously consisted only of the MazEF and RelBE superfamilies found in lactobacilli and bifidobacteria. To expand the database, we conducted an advanced search for type II TAS based on literature reports [8,19,20,21,22]. To search for literature sources, we used the following search engines and databases: Google, Google Scholar, Scopus and Medline. The primary keywords used were “toxin–antitoxin systems” and “toxin–antitoxin systems type II”. As a result, we found 18 additional type II TAS adding up to a total of 20 considering MazEF and RelBE, which were added to the catalog, and all of which are represented in Table 1.

To identify the main domains in the proteins of the TA systems reported in the literature (Table 1), we used Pfam (http://pfam.xfam.org/) [23] and UniPlot (http://www.uniprot.org/) databases. For each domain, we attributed its corresponding function as indicated in the Pfam database and retrieved its geninfo identifier (GI) number from the NCBI database.

After identifying all the domains, we blasted those with unknown function (DUF) against the NCBI database to find any possible homology with that of toxins and antitoxins. The maximum homology with TAS domains did not exceed 40%, which led us to exclude DUFs from further analysis. We also rejected the domains which belonged to antitoxins involved in binding to DNA: Arc, MetJ, OmegaRepress, parG, PSK trans fac, REGB T4, RepB RCR reg, RHH 1, RHH 3, RHH 4, RHH 5, RHH 7, SeqA N, TraY, VirC2, DndE, MatP C, Plasmid stab B, Repressor Mnt and RepB-RCR req. Pfam protein families of toxins and antitoxins used in this study are presented in Table 2. Each domain belonged to one of the following four Pfam clans (Table 2):

*Plasmid-antitox (CL0136)*—this clan includes mainly antitoxins participating in the preservation of plasmid-harboring bacteria.

*Met_repress (CL0057)*—this clan includes domains of only the antitoxins containing ribbon-helix-helix (RHH) motifs in the DNA-binding region.

*Ccdb_PemK (CL0624)*—this superfamily includes cell growth inhibitors and toxin components.

*AbrB (CL0132)*—this superfamily includes the DNA-binding domain of AbrB and the putative DNA-binding protein MraZ.

To conduct further analysis, we used the domains of toxin and antitoxin proteins from the two main superfamilies MazEF and RelBE and the family HicA-HicB (Table S1).

We assembled a gene catalog containing the retrieved GI numbers using a script written in the Python programming language and Biopython. This script allows the user to automatically open all folders with the initial GI numbers, save them, and, using Entrez.efetch, get the corresponding fasta file with proteins as an output. Further, we only selected the sequences corresponding to 55 of the most common genera of bacteria from the human GI tract [24]. We ruled out the repeated sequences (some of the domains belonged to a single TAS) using CD-HIT (http://weizhongli-lab.org/cd-hit/) software.

In addition, we created another catalog consisting of files from the GenBank database (gb files) containing information about the proteins and their coding regions, each of which are assigned a unique accession number. Next, we selected those sequences whose gb files contained information about the coding region (CDS). For each such sequence, we downloaded the whole genome of the corresponding bacterium, which was used to create a FASTA file containing characteristics of the bacterium and the nucleotide sequence of the gene. Following these steps, we were able to create a catalog consisting of 5299 nucleotide sequences. 

Since TA genes are prone to horizontal gene transfer, we had to exclude those localized on plasmids. To achieve this task, we created an additional catalog of all the plasmids submitted to the NCBI database, which numbered 51,988 sequences. Next, we identified the TA genes localized on plasmids using Blast software with the settings: query coverage > 80% and identity > 70%. This way we identified and deleted from the catalog 525 sequences containing TA genes, which were found in the genera *Anaerobaculum*, *Bryantella*, *Leuconostos*, *Mollicutes* and *Turicibacter*.

The final catalog consisted of 4239 nucleotide sequences belonging to 49 different genera, 489 species and 1346 strains (Table 3). The average length of genes reached 306 base pairs. The number of genes per species varied from one to three.

### 2.2. Clusterization of Type II TAS

**Figure 1:**
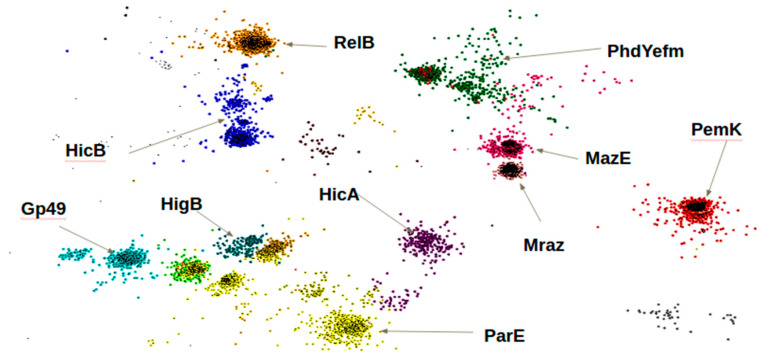
Two-dimensional model of the relative localization of TA protein families, constructed with CLANS software.

After creating a database of TAS, which fulfilled the inclusion criterion of the selected domains, we grouped the toxin and antitoxin proteins into families. It is known that the proteins of toxins and antitoxins inside the bacterial species share a significant identity of up to 98%. To verify the relatedness between proteins of divers species and genus in our database, we analyzed 4239 amino acid sequences using the CLANS software (http://www.eb.tuebingen.mpg.de/research/departments/proteinevolution/software/clans.html). Figure 1 shows a 2D model of localization of TA proteins relative to each other, constructed using the CLANS software dimensionality reduction. The figure illustrates the clusterization of the proteins in our catalog according to the Pfam protein families to which they belong. Each dot on the figure corresponds to a single amino acid sequence of a protein containing a domain, which was assigned a distinguishing color.

The figure illustrates the high sequence identity between proteins grouped into one family of toxin or antitoxin (Table 1). Some of the protein families such as Mraz and MazE are situated close to each other due to their high sequence similarity. The clusterization of proteins of the TA system according to the selected domains revealed the uniqueness of protein families as well as the differences between them.

### 2.3. Optimization of Control Parameters of the TAGMA Software on Metagenome Samples

We simulated nine metagenome samples with different numbers of reads, genera and species and genome size (Tables S2 and S3). We simulated reads with both similar and different genome sizes. For simulated reads we used DWGSIM (https://github.com/nh13/DWGSIM). The number of reads varied between 10^6^ and 5 × 10^7^ per sample. The genome coverage varied between three and seven reads per segment of the genome. The total number of species varied between 18 and 37.

TAGMA generates files containing information about markers, mapped genes and detected strains. To carry out our analysis, we opted for the files titled summary.txt and test_results_short.txt. The file summary.txt contains information about the identified strains, their uniqueness, their distinguishing markers, the overall number of markers and the marker coverage. The file test_results_short.txt contains information about the identified strains, their distinguishing markers, the marker coverage and the positions of significant single nucleotide polymorphism (SNP) unique to those markers.

As shown by the validation of the simulated data, TAGMA generated a high false positive rate (FPR). To readjust the software, we had to seek the optimal threshold values that allowed the identification of the maximum number of strains while maintaining a low level of false results. We chose the Jaccard index (JI) as an indicator of congruency between the input data used to simulate the metagenomes and the output data generated by TAGMA. As long as the number of false results is minimal, the higher the JI, the higher is the congruency between the input data and the results. We varied the parameters represented in Table 4 using a simulated genome sample using a script written in Python. 

We narrowed it down to the 12 most optimal thresholds (Table S4), which reflected the best ratio between a high JI and a low FPR. We calculated the JI and the FPR for the 12 thresholds (Tables S5 and S6). As can be inferred from Tables S5 and S6, the values of the JI and the FPR varied between the simulated metagenome samples depending on the threshold. To select the optimal threshold, we calculated the mean values of the JI and the FPR for each threshold (see Table S7).

As shown in Table S7, threshold 1 was deemed optimal because it generated the lowest FPR while maintaining an acceptable JI. The other parameters were:

1. Uniqueness = 1—only unique results (cannot be confused with others) were admissible.
2. Coverage = 170 b.p.—the minimal length in b.p. of the marker covering a gene.
3. Coverage = 98%—the minimal coverage rate for each gene.
4. Number of significant SNPs = 3—the minimal number of SNPs used for the mapping of a particular gene.
5. Threshold number of TAS = 5—the minimal required number of TAS used for the identification of a species.
6. Number of TAS in summary.txt = 1—the minimal required number of TAS used for the identification of a species.
7. Number of TAS in test_results_short.txt = 2—the minimal required number of TAS used for the identification of a species.

The JI and the FPR in the case of the optimal threshold are listed in Table S8. For some samples, the FPR was zero with a high JI index. Moreover, even though the JI index in the fourth and eighth samples was lowest, their error rate was low too. It is noteworthy that TAGMA detected all the species in the input file. Since the generated file included some noise, we had to set the optimal threshold to eliminate it as much as possible. Consequently, the set threshold excluded some of the valid species. Some sequences in the TAS database were only referred to the corresponding genus, which led to their exclusion from the results thereby lowering the JI.

### 2.4. Comparing TAGMA to MetaPhlAn2

The generated reads were simultaneously processed using MetaPlAn2. Table 5 shows that TAGMA surpassed MetaPlAn2 by generating results with a higher JI, both before and after adjusting the thresholds.

The most plausible result yielded by MetaPhlAn2 was in the case of the fourth sample (JI = 0.79). However, in the case of the eighth sample, the JI was lowest. As for TAGMA, it yielded maximal results in two cases before its thresholds and in six cases after adjusting them. As for the FPR of results generated by MetaPhlAn2, it was at its lowest in the case of the fourth sample. Before adjusting the thresholds, the results generated by TAGMA displayed a high FPR for all samples, including sample number four with a low JI (Table 6). To compare the efficiency of the software, we calculated the mean values for the JI and false positive (Table 7).

After adjusting the thresholds of TAGMA, it generated the highest mean JI of 0.70, which is an improvement of 0.06 over its mean JI before the adjustment and an improvement of 0.11 over MetaPhlAn. Moreover, the average FPR characteristic of TAGMA after the adjustment reached 32%, which exceeded the average FPR yielded by MetaPhlAn2 by 7%, an overall improvement of 18%.

### 2.5. Taxonomic Profiling of Metagenomes Using TAGMA

To validate the TAGMA (Toxin Antitoxin Genes for Metagenomes Analyses) software with the created database, we selected 20 metagenomes isolated from children aged between one and nine and living in the central region of Russia (see Materials and Methods, Table 8). TAGMA is a pipeline consisting of an existing published software and in-house scripts (https://github.com/LabGenMO/TAGMA). The algorithm first scans BLASTN alignments of TAS and identifies markers (substitutions and indels) that distinguish gene variants, identified by all to all BLASTN alignments. Then, it aligns the metagenomics reads against TAS genes using BowTie2. Finally, the software presents significant hits [18].

The samples were subjected to both whole genome sequencing (WGS) and 16S rRNA gene sequencing to obtain a better representation of the bacterial composition. The 16S rRNA gene sequencing data were analyzed using RDP software (http://rdp.cme.msu.edu/). The WGS data were analyzed with MetaPhlan2 [25], Kraken2 [26], Centrifuge [27] and TAGMA (Table S9). 16S rRNA sequencing allowed us to characterize the bacterial diversity of samples at the genus level. The WGS data revealed to us a fair quantitative representation of each genus and species in the metagenomes. Table S9 shows the bacterial diversity of metagenomes (over 0.01%).

After identifying the main genera, we analyzed the WGS data for species identification (Table S9). Compared to MetaPhlan2, Kraken2 and Centrifuge, TAGMA shows similar bacterial diversity at the species level within the 55 genera. Further we opted for MetaPhlAn2 for future comparison because it is the kernel of a large number of taxonomic classification software. Moreover, the TAGMA software was able to analyze the WGS data on the level of strains or groups of strains (Table 9 and Table S10). In those cases, when TAGMA failed to identify the strains, only the species or genus is shown. These results support the use of TAS II as markers for the phylogenetic profiling of the GM.

## 3. Discussion

The wide spread of TAS type II among bacteria has already been shown [13,28]. Further studies have shown that genes of TAS type II proteins are present in all investigated genomes [29,30]. This is also confirmed by data from the TADB database (https://bioinfo-mml.sjtu.edu.cn/TADB2/index.php). Some of the conclusions drawn in our previous study of TAS of lactobacilli and bifidobacteria were that (1) all the studied species had species-specific toxin and antitoxin genes and (2) distribution of almost any gene reveals some degree of species specificity. In other words, genes/proteins of TAS are suitable for species- and in some cases strain-level identification in lactobacilli and bifidobacteria isolated from the human GM [18]. In this study, we expanded the number of toxin and antitoxin proteins/genes and the list of analyzed bacteria to 489 species of 49 genera. The updated database of TA genes includes 4239 nucleotide sequences altogether.

Using the expanded database of type II TAS, we validated the TAGMA software’s capacity of analyzing the taxonomic composition of the HGM. To achieve this task, we carried out WGS and targeted 16S rRNA sequencing of 20 fecal samples isolated from humans. 16S rRNA sequencing allowed us to characterize the bacterial diversity of the samples at the genus level. Contrastingly, WGS turned out to be more informative since it allowed us to characterize the diversity of the samples at the species level. Further data analysis carried out using TAGMA revealed that the vast majority of the bacteria identified in the metagenomes were identical to those identified using MetaPhlan2, Kraken2, Centrifuge and RDP. These results prove that genes encoding type II TAS can be used as functional markers for computer-assisted species characterization of the human GM. Moreover, analysis of WGS data revealed species, which, unlike MetaPhlan2, TAGMA succeeded in identifying.

Not only did TAGMA surpass other programs in species identification, it also allowed us to identify single strains or groups of strains. Individual differences between the GM and their impact on their hosts are determined by the microbiome composition not only at the level of phyla or species but at the strain level too. The characteristics of a strain of an individual microbiota are stable throughout its life with differences consistently lower than differences between people [31]. The strain specificity of the microbiota determines the functional features of an individual’s microbiota and the propensity for certain diseases [32], as well as the possibility of correction of gut immune phenotype [33]. Identification of strain composition in microbiota is necessary for a personalized scheme of prevention and treatment of human diseases.

Based on our results of 16S sequence analysis, the genus *Blautia* is predominant in the samples, whereas according to WGS and MetaPhlan2, this genus is not present in all samples. One possible explanation is the absence of markers of this genus and its species in MetaPhlan2. To be more specific, analysis carried out using TAGMA allowed us to detect strains belonging to the groups *B. obeum* A2-162, *B. obeum* ATCC 29174 and *B. hydrogenotrophica* DSM 10507. It was noted previously that the prevalent bacterial genus, *Faecalibacterium*, was represented by a single species—*F. prausnitzii*. Nevertheless, the TAGMA software detected multiple strains of this species: *F. prausnitzii* A2-165, *F. prausnitzii* L2-6 2, *F. prausnitzii* M21/2 and *F. prausnitzii* SL3/3.

Bifidobacteria are among the first anaerobic commensal microorganisms to colonize the intestines of children—they are highly abundant in two-thirds of newborn infants in their first week of life [34]. As the predominant component of infants’ microbiota, bifidobacteria promote the development of the immune system of humans in the postnatal period. As infants grow up, the percentage of bifidobacteria in their GM drops to 11% in children between one and four years of age and to 3% in adults [35]. According to the literature, *B.longum* is the most common species of bifidobacteria in the human microbiome, which is consistent with the results obtained using TAGMA revealing many strains belonging to this species.

## 4. Conclusions

Our results enable us to consider the TAGMA program and TAS as highly effective tools for species- and in some cases strain-level profiling of single genomes as well as metagenomes. We varied seven parameters, thereby testing 10,000,000 different combinations. After narrowing it down to 12 combinations, we chose the one yielding the lowest FPR and the highest JI. After the validation, the FPR amounted to 8%, which is an improvement of 17% over MetaPhlAn2. Thus, TAGMA proved to be more accurate after optimizing its parameters. While MetaPhlan2 uses up to a million genomic markers to determine the species in metagenomes, TAGMA does not need more than 10 markers to identify one species. In this study, our search software only detected the main 55 bacterial genera inhabiting the GM that were incorporated into its catalog. The software proved to be a useful tool for identifying strains of these bacteria. We intend, however, to expand our database to include bacterial strains inhabiting other biotopes of the human body. Nevertheless, the most comprehensive information on species composition in metagenomes is revealed using several programs.

## 5. Materials and Methods

### 5.1. Metagenomic Analysis Software

TA genes, which are part of a pipeline consisting of existing published software and in-house scripts (https://github.com/LabGenMO/TAGMA) served as a reference for metagenomic analyses [18].

### 5.2. Creation of the Catalog of TA Genes

The domains of TAS were taken from the databases Pfam and UniProt. The catalogs of amino acid and nucleotide sequences were taken from the NCBI database. To download the databases, we used scripts written in Python and Biopython (http://biopython.org/). Duplicate sequences generated by the domain database Pfam were removed with CD-HIT.

### 5.3. Exclusion of Plasmid Genes

A total of 49.353 nucleotide sequences of plasmids were downloaded from NCBI by using the entry query “NUCCORE:plasmid[Title].” Nucleotide sequences of TAS genes were mapped using the BLASTN program against the plasmid sequences. The hits were filtered with identity threshold values of 80%. If a plasmid hit was located for a TAS gene hit, we assumed that this TAS gene could be transferred by plasmids. 

### 5.4. CLANS

The program (http://www.eb.tuebingen.mpg.de/research/departments/protein-evolution/software/clans.html) allows aminoacid sequences to be clustered based on the homology between them. Each sequence is BLASTed against another, allowing the determination of the homology between them, after which these numbers are translated into forces of attraction and repulsion. The identical sequences are attracted to one another, grouping into clusters.

### 5.5. In-House Metagenome Characterization

We used gut metagenomes (fecal samples) isolated from healthy children aged between one to three years old living in the central region of Russia. All 20 metagenomes have been deposited in GenBank (NCBI) (Table 8).

### 5.6. Analysis of 16S rRNA Gene Amplicon Sequences

Amplicon libraries targeting the 16S rRNA gene V3-V4 regions were prepared following 16S Metagenomic Sequencing Library Preparation protocols. (https://support.illumina.com/content/dam/illuminamarketing/documents/products/appnotes/16S-Metagenomic-Library-Prep-Guide.pdf). Sequencing was performed on the Illumina MiSeq platform (Illumina, San Diego, CA, USA) following the manufacturer’s specifications. We used trimmomatic v0.3 software for read trimming and FastQC v.0.10.128 for quality control. Raw sequence data were processed and analyzed using QIIME (Quantitative Insights Into Microbial Ecology, Version 1.9.1) software and Ribosomal Database Project (RDP) http://rdp.cme.msu.edu/.

### 5.7. Fragment DNA and Library Preparation for Illumina Sequencing

First, 300 ng of DNA was sheared using sonication with a Covaris S220 system (Covaris, Woburn, Massachusetts, MA, USA). The final size of fragmented DNA samples as assessed in an Agilent 2100 Bioanalyzer (Agilent Technologies, Santa Clara, CA, US) was in the range 400–500 bp. Paired-end libraries were prepared following the manufacturer’s specifications using a NEBNext Ultra II DNA Library Prep Kit (New England Biolabs, Ipswich, MA, USA). The libraries were indexed with NEBNext Multiplex Oligos for Illumina (96 Index Primers) (New England Biolabs, Ipswich, MA, USA) kits. The libraries’ size distribution and quality were assessed using a high sensitivity DNA chip (Agilent Technologies, Santa Clara, CA, US). Libraries were subsequently quantified using a Quant-iT DNA Assay Kit, High Sensitivity (Thermo Scientific, Walthman, MA, USA). Sequencing was performed on the HiSeq 2500 platform (Illumina, San Diego, CA, USA) according to the manufacturer’s recommendations using the following reagent kits: HiSeq PE Cluster Kit v2, HiSeq SBS Kit v2 (250 cycles), HiSeq Rapid PE FlowCell v2 and a 2% PhiX spike-in control.

## Figures and Tables

**Table 1:** Type II TAS used in this study.

System	Toxin	Antitoxin
BrnT/BrnA	BrnT	BrnA
Ccd	CcdB	CcdA
HicAB	HicA	HicB
HigBA	HigB	HigA
HipAB	HipA	HipB
MazEF	MazF	MazE
MqsR/MqsA	MqsR	MqsA
Omega-Epsilon-Zeta (ω-ε-ζ)	Zeta	Epsilon
ParDE	ParE	ParD
PezA/Pez	PezT	PezA
Phd/Doc	Doc	Phd
PhoAT/PhoH2	PhoH2	PhoAT
RelBE	RelE	RelB
VapBC	VapC	VapB
YafQ/DinJ	YafQ	DinJ
YoeB/YefM	YoeB	YefM
RatA/RatB	RatA	RatB
FitB/FitA	FitB	FitA
Kid/Kis	Kid	Kis
AtaT/AtaR	ataT	ataR

**Table 2:** Pfam protein families of toxins and antitoxins used in this study.

Pfam Clan	Pfam Protein Family of Toxins	Pfam Protein Family of Antitoxins
***Plasmid-antitox (CL0136)***	RelE (PF06296)	PhdYeFM_antitox (PF02604)
	Gp49 (PF05973)	RelB (PF04221)
	HigB-like_toxin (PF05015)	PHD_like (PF12910)
	ParE-like_toxin (PF15781)	
	ParE_toxin (PF05016)	
	YafQ_toxin (PF15738)	
	MqsR_toxin (PF15723)	
	BrnT-toxin (PF04365)	
	YoeB_toxin (PF06769)	
***Met_repress (CL0057)***	HicA_toxin (PF07927)	CcdA (PF07362)
		BrnA_antitoxin (PF14384)
		CopG_antitoxin (PF12441)
		HicB (PF05534)
		HicB-like_2 (PF15970)
		HicB_lk_antitox (PF15919)
		ParD (PF09386)
		ParD_antitoxin (PF03693)
		ParD_like (PF11903)
		VapB_antitoxin (PF09957)
***Ccdb_PemK (CL0624)***	PemK_toxin (PF02452)	
	CcdB (PF01845)	
***AbrB (CL0132)***		MazE_antitoxin (PF04014)
		MraZ (PF02381)
		PrlF_antitoxin (PF15937)

**Table 3:** The number of genera, species and strains in the final catalog.

No.	Genus	N of Species	N of Strains
1	*Acidaminococcus*	1	5
2	*Actinomyces*	13	22
3	*Akkermansia*	2	4
4	*Alistipes*	1	1
5	*Anaerococcus*	4	5
6	*Anaerofustis*	1	1
7	*Anaerostipes*	2	6
8	*Anaerotruncus*	1	2
9	*Bacteroides*	19	64
10	*Bifidobacterium*	33	79
11	*Blautia*	3	9
12	*Butyrivibrio*	4	10
13	*Catenibacterium*	1	2
14	*Clostridium*	42	109
15	*Collinsella*	4	5
16	*Coprococcus*	3	5
17	*Desulfovibrio*	11	15
18	*Dialister*	3	4
19	*Dorea*	2	7
20	*Eggerthella*	1	2
21	*Enterobacter*	6	34
22	*Enterococcus*	27	82
23	*Escherichia*	4	106
24	*Eubacterium*	18	31
25	*Faecalibacterium*	2	9
26	*Fusobacterium*	5	29
27	*Gordonibacter*	1	1
28	*Helicobacter*	13	43
29	*Holdemania*	1	1
30	*Klebsiella*	4	58
31	*Lactobacillus*	137	225
32	*Listeria*	13	18
33	*Megamonas*	1	1
34	*Megasphaera*	2	3
35	*Methanobrevibacter*	6	6
36	*Mitsuokella*	1	1
37	*Odoribacter*	2	2
38	*Oxalobacter*	1	2
39	*Parabacteroides*	4	9
40	*Pediococcus*	7	14
41	*Prevotella*	15	26
42	*Proteus*	4	10
43	*Providencia*	5	15
44	*Roseburia*	4	12
45	*Ruminococcus*	9	22
46	*Streptococcus*	31	208
47	*Subdoligranulum*	1	1
48	*Veillonella*	4	8
49	*Weissella*	10	12

**Table 4:** Value ranges of the selected parameters.

Parameter Number	Parameter	Value Range
1	Uniqueness	1–15
2	Coverage, b.p.	30–280
3	Coverage, %	40–100
4	Number of significant SNPs	0–20
5	Threshold number of TAS	2–15
6	Number of TAS in summary.txt	1–10
7	Number of TAS in test_results_short.txt	1–10

**Table 5:** Comparison of the mean Jaccard index (JI) of nine samples processed using MetaPhlAn2 and TAGMA before and after adjusting the thresholds.

	Samples	1	2	3	4	6	7	8	9
Software									
MetaPhlAn2		0.58	0.55	0.75	0.79	0.59	0.62	0.24	0.58
TAGMA (before adjustment)		0.45	0.73	0.74	0.56	0.75	0.71	0.73	0.47
TAGMA (after adjustment)		0.66	0.88	0.80	0.52	0.79	0.77	0.53	0.68

**Table 6:** Comparison of the false positive rate (FPR) of nine samples processed using MetaPhlAn2 and TAGMA before and after adjusting the thresholds.

	Samples	1	2	3	4	6	7	8	9
Software									
MetaPhlAn2		29.73	27.27	14.29	9.52	24.00	30.30	35.71	29.73
TAGMA (before adjustment)		53.03	25.00	20.69	33.33	22.86	28.57	25.00	50.79
TAGMA (after adjustment)		13.79	0.00	0.00	27.27	0.00	4.00	4.76	10.71

**Table 7:** Comparison of the mean JI and the FPR (%) of the results generated using MetaPhlAn2 and TAGMA before and after adjusting the thresholds.

	Indicators	Mean JI	Mean Standard Error for JI	Mean FPR (%)	Standard Error for the Mean Positive Rate (%)
Software					
MetaPhlAn2		0.59	0.06	25.07	3.13
TAGMA (before adjustment)		0.64	0.05	32.41	4.47
TAGMA (after adjustment)		0.70	0.05	7.57	3.35

**Table 9:** The number of species and strains in sample A1 (species over 1%).

Sample	Species	N Species MetaPhlan2	N Species TAGMA	N Strain TAGMA
A1	*Alistipes*	1	1	1
	*Bacteroides*	4	3	7
	*Barnesiella*	1	-	-
	*Bifidobacterium*	2	5	9
	*Blautia*	-	1	1
	*Clostridium*	-	5	5
	*Coprococcus*	-	1	1
	*Dialister*	1	1	2
	*Dorea*	-	1	1
	*Eubacterium*	-	1	1
	*Faecalibacterium*	1	1	3
	*Megamonas*	1	1	1
	*Parabacteroides*	1	1	1
	*Roseburia*	-	2	2
	*Prevotella*	1	-	-
	*Ruminococcus*	1	1	1
	*Streptococcus*	1	2	3

**Table 8:** Description of metagenomes.

Accession No.	Reference in the Article	Description
SRX5327357	A1	male, 3 years old
SRX5327356	A2	3 years old
SRX5327362	A3	1 year old
SRX5327358	A4	4 years old
SRX5327344	A5	male, 3 years old
SRX5327353	A6	male, 7 years old
SRX5327352	A7	female, 9 years old
SRX5327351	A8	male, 7 years old
SRX5327350	A9	female, 3 years old
SRX5327347	A10	male, 9 years old
SRX5327345	A11	male, 4 years old
SRX5327355	A12	male, 3 years old
SRX5327354	A13	male, 4 years old
SRX5327360	A14	male, 8 years old
SRX5327359	A15	male, 3 years old
SRX5327349	B1	infant (0–1 year)
SRX5327346	B2	infant (0–1 year)
SRX5327343	B3	infant (0–1 year)
SRX5327348	B4	infant (0–1 year)
SRX5327361	B5	infant (0–1 year)

## Supplementary Materials

The following are available online at https://www.mdpi.com/2072-6651/12/6/388/s1, Table S1: Domains from the Pfam database, Table S2: Characteristics of the metagenome samples listed in increasing numerical order, Table S3: Simulated metagenomes, Table S4: A representation of calculated parameters for each threshold, Table S5: The JI in the case of each threshold calculated for nine samples, Table S6: The FPR (%) in the case of each threshold calculated for nine samples, Table S7: The mean JI and FPR (%) in the case of each threshold calculated for nine samples, Table S8: JI and FPR (%) yielded by the optimal the threshold calculated for nine samples, Table S9: Comparative analysis of metagenomes, Table S10: Species and strains diversity in the sample.
