# Supplementary material for: Toxin-Antitoxin Systems: A Tool for Taxonomic Analysis of Human Intestinal Microbiota
Source: Toxins (Basel). 2020 Jun 12;12(6):388. doi: 10.3390/toxins12060388 (PMC7354421; doi:10.3390/toxins12060388)
Supplement: Supplementary file 1 [file toxins-12-00388-s001.zip › toxins-811256 supplementary proof done/toxins-811256 supplementary.docx]

Supplementary Materials: Toxin-Antitoxin Systems: A Tool for Taxonomic Analysis of Human Intestinal Microbiota

Ksenia M. Klimina, Viktoriya N. Voroshilova, Elena U. Poluektova, Vladimir A. Veselovsky,
Roman A. Yunes, Aleksey S. Kovtun, Anna V. Kudryavtseva, Artem S. Kasianov and
Valery N. Danilenko

**Table 1.** Domains from the Pfam database.

| **Toxins** | **Antitoxins** |
| --- | --- |
| **Domains from the RelBE Family** | |
| RelE | CcdA |
| Gp49 | BrnA_antitoxin |
| HigB-like_toxin | MqsA |
| ParE-like_toxin | CopG_antitoxin |
| ParE_toxin | HicB |
| YafQ_toxin | HicB-like_2 |
| MqsR_toxin | HicB_lk_antitox |
| BrnT-toxin | ParD |
| YoeB_toxin | ParD_antitoxin |
|  | ParD_like |
|  | PhdYeFM_antitox |
|  | RelB |
|  | PHD_like |
|  | VapB_antitoxin |
|  | VAPB_antitox |
| **Domains from the MazEF и HicAB family** | |
| PemK_toxin | MazE_antitoxin |
| [CcdB](http://pfam.xfam.org/family/PF01845) | [MraZ](http://pfam.xfam.org/family/PF02381) |
|  | PrlF_antitoxin |
| HicA_toxin |  |

**Table 2.** Characteristics of the metagenome samples listed in increasing numerical order.

| **Sample Number** | **The Number of reads, 10^6^ b.p.** | **The Number of Species** | **The Genome Size Range, 10^6^ b.p.** |
| --- | --- | --- | --- |
| **1** | 50 | 34 | 2,6 |
| **2** | 1 | 25 | 3,4 |
| **3** | 1 | 25 | 2,3 |
| **4** | 1 | 25 | 4,8 |
| **5** | 1 | 18 | 2,3 |
| **6** | 1 | 28 | 2,4 |
| **7** | 1 | 30 | 2,4 |
| **8** | 1 | 37 | 1,6 |
| **9** | 10 | 34 | 2,6 |

**Table 3.** Simulated metagenomes.

| **1** | **2** | **3** | **4** | **5** | **6** | **7** | **8** | **9** |
| --- | --- | --- | --- | --- | --- | --- | --- | --- |
| *Prevotella dentalis* | *Actinomyces naeslundii DNA* | *Acidaminococcus fermentans DSM 20731* | *Bacteroides cellulosilyticus* | *Acidaminococcus fermentans DSM 20731* | *Actinomyces naeslundii* | *Acidaminococcus fermentans DSM 20731* | *Actinomyces georgiae F0490* | *Prevotella dentalis* |
| *Parabacteroides johnsonii* | *Akkermansia glycaniphila* | *Actinomyces odontolyticus ATCC 17982* | *Bacteroides dorei* | *Actinomyces odontolyticus ATCC 17982* | *Akkermansia glycaniphila* | *Actinomyces naeslundii* | *Actinomyces naeslundii str. Howell 279* | *Parabacteroides johnsonii* |
| *Enterococcus faecalis* | *Bifidobacterium scardovii JCM 12489 = DSM 13734* | *Actinomyces radingae* | *Bacteroides salanitronis DSM 18170* | *Bacteroides vulgatus strain mpk* | *Bifidobacterium scardovii JCM 12489 = DSM 13734* | *Akkermansia muciniphila ATCC BAA-835* | *Anaerostipes caccae DSM 14662* | *Enterococcus faecalis* |
| *Eubacterium callanderi* | *Butyrivibrio proteoclasticus B316* | *Akkermansia muciniphila ATCC BAA-835* | *Bacteroides vulgatus* | *Bacteroides thetaiotaomicron strain 7330* | *Butyrivibrio proteoclasticus B316* | *Anaerofustis stercorihominis DSM 17244* | *Bacteroides cellulosilyticus CL02T12C19* | *Eubacterium callanderi* |
| *Eggerthella lenta* | *Clostridium kluyveri NBRC 12016* | *Bifidobacterium thermophilum RBL67* | *Bacteroides ovatus V975* | *Bifidobacterium longum strain 35624* | *Clostridium kluyveri NBRC 12016]* | *Bacteroides sp.* | *Bifidobacterium longum subsp. infantis ATCC 15697 = JCM 1222 = DSM 20088* | *Eggerthella lenta* |
| *Megasphaera elsdenii* | *Clostridium botulinum A3 str. Loch Maree* | *Bifidobacterium bifidum* | *Clostridium sp.* | *Bifidobacterium breve strain BR3* | *Clostridium botulinum A3 str. Loch Maree* | *Bifidobacterium thermophilum RBL67* | *Bifidobacterium adolescentis* | *Megasphaera elsdenii* |
| *Enterobacter cloacae* | *Clostridium acetobutylicum EA 2018* | *Bifidobacterium adolescentis ATCC 15703* | *Clostridium saccharoperbutylacetonicum N1-4(HMT)* | *Clostridium botulinum strain CDC_69094* | *Clostridium acetobutylicum EA 2018* | *Clostridium sp.* | *Butyrivibrio proteoclasticus* | *Enterobacter cloacae* |
| *Odoribacter splanchnicus* | *Clostridium perfringens* | *Bifidobacterium dentium ATCC 27678* | *Clostridium pasteurianum BC1* | *Klebsiella pneumoniae str. Kp52.145* | *Clostridium perfringens* | *Desulfovibrio africanus str. Walvis Bay* | *Clostridium ragsdalei P11* | *Odoribacter splanchnicus* |
| *Ruminococcus flavefaciens* | *Desulfovibrio vulgaris RCH1* | *Bifidobacterium longum subsp. infantis ATCC 15697 = JCM 1222 = DSM 20088* | *Clostridium saccharobutylicum DSM 13864* | *Lactobacillus reuteri strain ZLR003* | *Desulfovibrio vulgaris RCH1* | *Dialister invisus DSM 15470* | *Collinsella stercoris DSM 13279* | *Ruminococcus flavefaciens* |
| *Desulfovibrio magneticus* | *Desulfovibrio alaskensis G20* | *Bifidobacterium asteroides PRL2011* | *Desulfovibrio africanus str. Walvis Bay* | *Lactobacillus brevis strain NPS-QW-145* | *Desulfovibrio alaskensis G20* | *Dorea formicigenerans ATCC 27755* | *Coprococcus catus GD/7* | *Desulfovibrio magneticus* |
| *Bifidobacterium scardovii* | *Desulfovibrio hydrothermalis AM13 = DSM 14728* | *Bifidobacterium breve JCM 7019* | *Desulfovibrio magneticus RS-1* | *Lactobacillus fermentum strain SNUV175* | *Desulfovibrio hydrothermalis AM13 = DSM 14728* | *Eggerthella lenta DSM 2243* | *Desulfovibrio vulgaris RCH1* | *Bifidobacterium scardovii* |
| *Subdoligranulum variabile* | *Eggerthella lenta DSM 2243* | *Clostridium sp.* | *Desulfovibrio sp.* | *Prevotella intermedia* | *Eggerthella lenta DSM 2243* | *Enterobacter cloacae* | *Dorea formicigenerans ATCC 27755* | *Subdoligranulum variabile* |
| *Helicobacter pullorum* | *Eggerthella sp.* | *Clostridium botulinum B2 128* | *Enterobacter cloacae* | *Providencia rettgeri strain RB151* | *Eggerthella sp.* | *Enterococcus mundtii QU 25* | *Enterobacter hormaechei ATCC 49162* | *Helicobacter pullorum* |
| *Blautia obeum* | *Enterobacter sp.* | *Clostridium sporogenes ATCC 15579* | *Enterobacter asburiae* | *Streptococcus pneumoniae strain SWU02* | *Enterobacter sp.* | *Escherichia fergusonii ATCC 35469* | *Enterococcus villorum ATCC 700913* | *Blautia obeum* |
| *Fusobacterium nucleatum* | *Enterococcus mundtii QU 25* | *Clostridium perfringens SM101* | *Enterobacter sp.* | *Streptococcus pyogenes MGAS315* | *Enterococcus mundtii QU 25* | *Eubacterium limosum KIST612* | *Escherichia coli 1-110-08_S1_C2* | *Fusobacterium nucleatum* |
| *Clostridium perfringens* | *Enterococcus rotai* | *Clostridium novyi NT* | *Escherichia fergusonii ATCC 35469* | *Streptococcus mutans UA159* | *Enterococcus rotai strain LMG 26678* | *Fusobacterium nucleatum subsp. animalis* | *Eubacterium barkeri* | *Clostridium perfringens* |
| *Clostridium sporogenes* | *Enterococcus silesiacus* | *Desulfovibrio piger* | *Escherichia coli S88* | *Enterococcus faecalis strain KB1* | *Enterococcus silesiacus strain LMG 23085* | *Helicobacter bizzozeronii CIII-1* | *Faecalibacterium prausnitzii L2-6* | *Clostridium sporogenes* |
| *Pediococcus acidilactici* | *Eubacterium rectale ATCC 33656* | *Desulfovibrio sp.* | *Escherichia albertii* | *Escherichia coli CFT073* | *Eubacterium rectale ATCC 33656* | *Klebsiella pneumoniae UCI 18* | *Fusobacterium sp.* | *Pediococcus acidilactici* |
| *Bacteroides intestinalis* | *Lactobacillus casei W56* | *Enterococcus faecalis AR01/DG* | *Eubacterium limosum KIST612* |  | *Lactobacillus casei W56* | *Lactobacillus casei W56* | *Helicobacter fennelliae MRY12-0050* | *Bacteroides intestinalis* |
| *Bacteroides ovatus* | *Lactobacillus rhamnosus GG* | *Escherichia coli 3003* | *Klebsiella michiganensis KCTC 1686* |  | *Lactobacillus rhamnosus GG* | *Listeria grayi DSM 20601* | *Klebsiella pneumoniae* | *Bacteroides ovatus* |
| *Eubacterium ramulus* | *Lactobacillus pentosus IG1* | *Fusobacterium nucleatum subsp. animalis* | *Klebsiella pneumoniae 342* |  | *Lactobacillus pentosus IG1* | *Methanobrevibacter millerae* | *Lactobacillus nodensis DSM 19682 = JCM 14932 = NBRC 107160* | *Eubacterium ramulus* |
| *Butyrivibrio fibrisolvens* | *Lactobacillus oris* | *Helicobacter cinaedi PAGU611* | *Klebsiella oxytoca* |  | *Lactobacillus oris strain J-1* | *Oxalobacter formigenes OXCC13* | *Lactobacillus reuteri* | *Butyrivibrio fibrisolvens* |
| *Streptococcus henryi* | *Lactobacillus koreensis* | *Klebsiella pneumoniae UCICRE 2* | *Proteus mirabilis HI4320* |  | *Lactobacillus koreensis strain 26-25* | *Parabacteroides goldsteinii CL02T12C30* | *Lactobacillus paracasei subsp. paracasei Lpp41* | *Streptococcus henryi* |
| *Escherichia vulneris* | *Lactobacillus plantarum* | *Lactobacillus acidipiscis* | *Providencia rettgeri* |  | *Lactobacillus plantarum strain LZ95* | *Pediococcus damnosus* | *Lactobacillus iners LactinV 11V1-d* | *Escherichia vulneris* |
| *Lactobacillus farraginis* | *Listeria monocytogenes str. Scott A* | *Lactobacillus buchneri NRRL B-30929* | *Providencia stuartii MRSN 2154* |  | *Listeria monocytogenes str. Scott A* | *Prevotella melaninogenica ATCC 25845* | *Listeria seeligeri FSL N1-067* | *Lactobacillus farraginis* |
| *Weissella cibaria* |  |  |  |  | *Prevotella multisaccharivorax DSM 17128* | *Proteus mirabilis BB2000* | *Parabacteroides merdae ATCC 43184* | *Weissella cibaria* |
| *Lactobacillus intestinalis* |  |  |  |  | *Proteus mirabilis BB2000* | *Providencia rettgeri* | *Prevotella sp.* | *Lactobacillus intestinalis* |
| *Roseburia hominis* |  |  |  |  | *Ruminococcus albus 7 = DSM 20455* | *Streptococcus thermophilus* | *Prevotella intermedia* | *Roseburia hominis* |
| *Proteus mirabilis* |  |  |  |  |  | *Veillonella parvula DSM 2008* | *Proteus mirabilis* | *Proteus mirabilis* |
| *Faecalibacterium prausnitzii* |  |  |  |  |  | *Weissella koreensis KACC 15510* | *Providencia rettgeri* | *Faecalibacterium prausnitzii* |
| *Oxalobacter formigenes* |  |  |  |  |  |  | *Roseburia inulinivorans DSM 16841* | *Oxalobacter formigenes* |
| *Veillonella atypica* |  |  |  |  |  |  | *Roseburia intestinalis XB6B4* | *Veillonella atypica* |
| *Streptococcus agalactiae* |  |  |  |  |  |  | *Ruminococcus torques L2-14* | *Streptococcus agalactiae* |
| *Dorea formicigenerans* |  |  |  |  |  |  | *Streptococcus thermophilus TH1477* | *Dorea formicigenerans* |
|  |  |  |  |  |  |  | *Streptococcus pneumoniae* |  |
|  |  |  |  |  |  |  | *Veillonella dispar ATCC 17748* |  |
|  |  |  |  |  |  |  | *Weissella cibaria* |  |

**Table 4.** A representation of calculated parameters for each threshold.

| **Parameters**  **Thresholds** | **Uniqueness** | **Coverage, b.p.** | **Coverage, %** | **Number of Significant SNPs** | **Threshold Number of TAS** | **Number of TAS in Summary.txt** | **Number of TAS in Test_Results_Short.txt** |
| --- | --- | --- | --- | --- | --- | --- | --- |
| **1** | 1 | 150 | 98 | 3 | 6 | 1 | 2 |
| **2** | 1 | 160 | 98 | 3 | 6 | 1 | 2 |
| **3** | 1 | 170 | 98 | 3 | 5 | 1 | 2 |
| **4** | 1 | 180 | 88 | 3 | 5 | 2 | 1 |
| **5** | 1 | 190 | 98 | 7 | 5 | 1 | 1 |
| **6** | 1 | 180 | 90 | 4 | 9 | 1 | 1 |
| **7** | 1 | 180 | 95 | 4 | 9 | 1 | 1 |
| **8** | 1 | 190 | 80 | 6 | 9 | 2 | 1 |
| **9** | 1 | 190 | 90 | 6 | 9 | 1 | 1 |
| **10** | 1 | 190 | 95 | 6 | 9 | 1 | 1 |
| **11** | 1 | 150 | 98 | 3 | 6 | 1 | 2 |
| **12** | 1 | 160 | 98 | 3 | 6 | 1 | 2 |

**Table 5.** The JI in the case of each threshold calculated for nine samples.

| **Samples**  **Thresholds** | **1** | **2** | **3** | **4** | **5** | **6** | **7** | **8** | **9** |
| --- | --- | --- | --- | --- | --- | --- | --- | --- | --- |
| **1** | 0.66 | 0.88 | 0.80 | 0.52 | 0.70 | 0.79 | 0.77 | 0.53 | 0.68 |
| **2** | 0.65 | 0.79 | 0.81 | 0.59 | 0.75 | 0.81 | 0.79 | 0.68 | 0.64 |
| **3** | 0.66 | 0.88 | 0.80 | 0.52 | 0.70 | 0.76 | 0.74 | 0.51 | 0.66 |
| **4** | 0.66 | 0.88 | 0.80 | 0.52 | 0.70 | 0.79 | 0.74 | 0.49 | 0.68 |
| **5** | 0.66 | 0.88 | 0.80 | 0.52 | 0.70 | 0.79 | 0.74 | 0.47 | 0.68 |
| **6** | 0.65 | 0.79 | 0.74 | 0.59 | 0.75 | 0.83 | 0.81 | 0.64 | 0.64 |
| **7** | 0.63 | 0.79 | 0.73 | 0.56 | 0.70 | 0.73 | 0.78 | 0.68 | 0.63 |
| **8** | 0.60 | 0.79 | 0.78 | 0.51 | 0.75 | 0.84 | 0.76 | 0.72 | 0.61 |
| **9** | 0.61 | 0.82 | 0.81 | 0.51 | 0.75 | 0.77 | 0.76 | 0.74 | 0.62 |
| **10** | 0.59 | 0.85 | 0.81 | 0.50 | 0.80 | 0.84 | 0.69 | 0.68 | 0.61 |
| **11** | 0.59 | 0.82 | 0.81 | 0.51 | 0.75 | 0.84 | 0.74 | 0.70 | 0.60 |
| **12** | 0.60 | 0.82 | 0.81 | 0.51 | 0.75 | 0.77 | 0.74 | 0.71 | 0.61 |

**Table 6.** The FPR (%) in the case of each threshold calculated for nine samples.

| **Samples**  **Thresholds** | **1** | **2** | **3** | **4** | **5** | **6** | **7** | **8** | **9** |
| --- | --- | --- | --- | --- | --- | --- | --- | --- | --- |
| **1** | 13.79 | 0.00 | 0.00 | 27.27 | 12.50 | 0.00 | 4.00 | 4.76 | 10.71 |
| **2** | 18.75 | 12.00 | 8.33 | 26.92 | 11.76 | 10.71 | 12.90 | 12.50 | 16.67 |
| **3** | 13.79 | 0.00 | 0.00 | 27.27 | 12.50 | 4.35 | 4.17 | 9.09 | 13.79 |
| **4** | 13.79 | 0.00 | 0.00 | 27.27 | 12.50 | 0.00 | 4.17 | 9.52 | 10.71 |
| **5** | 13.79 | 0.00 | 0.00 | 27.27 | 12.50 | 0.00 | 4.17 | 5.26 | 10.71 |
| **6** | 18.75 | 12.00 | 9.09 | 26.92 | 11.76 | 7.41 | 7.14 | 15.63 | 16.67 |
| **7** | 21.21 | 12.00 | 5.00 | 28.00 | 12.50 | 8.33 | 7.41 | 12.50 | 21.21 |
| **8** | 31.71 | 14.81 | 8.70 | 35.71 | 11.76 | 10.34 | 13.33 | 16.22 | 30.00 |
| **9** | 30.00 | 11.54 | 4.55 | 35.71 | 11.76 | 11.11 | 13.33 | 13.89 | 28.21 |
| **10** | 30.77 | 8.00 | 4.55 | 34.62 | 11.11 | 10.34 | 19.35 | 18.92 | 27.03 |
| **11** | 30.77 | 11.54 | 4.55 | 35.71 | 11.76 | 10.34 | 13.79 | 16.67 | 28.95 |
| **12** | 28.95 | 11.54 | 4.55 | 35.71 | 11.76 | 11.11 | 13.79 | 14.29 | 27.03 |

**Table 7.** The mean JI and FPR (%) in the case of each threshold calculated for nine samples.

| **Indicators**  **Threshold Value** | **Mean JI** | **Mean Standard Error for JI** | **Mean FSP (%)** | **Standard Error for the Mean Positive Rate (%)** |
| --- | --- | --- | --- | --- |
| **1** | 0.70 | 0.04 | 8.12 | 3.01 |
| **2** | 0.72 | 0.03 | 14.51 | 1.86 |
| **3** | 0.69 | 0.04 | 9.44 | 2.88 |
| **4** | 0.69 | 0.04 | 8.66 | 2.98 |
| **5** | 0.69 | 0.04 | 8.19 | 3.00 |
| **6** | 0.72 | 0.03 | 13.93 | 2.12 |
| **7** | 0.69 | 0.02 | 14.24 | 2.54 |
| **8** | 0.71 | 0.04 | 19.18 | 3.44 |
| **9** | 0.71 | 0.03 | 17.79 | 3.55 |
| **10** | 0.71 | 0.04 | 18.30 | 3.55 |
| **11** | 0.71 | 0.04 | 18.23 | 3.61 |
| **12** | 0.70 | 0.03 | 17.64 | 3.45 |

**Table 8.** JI and FPR (%) yielded by the optimal the threshold calculated for nine samples.

| **Samples**  **Indicators** | **1** | **2** | **3** | **4** | **5** | **6** | **7** | **8** | **9** |
| --- | --- | --- | --- | --- | --- | --- | --- | --- | --- |
| **JI** | 0.66 | 0.88 | 0.80 | 0.52 | 0.70 | 0.79 | 0.77 | 0.53 | 0.68 |
| FPR**, %** | 13.79 | 0.00 | 0.00 | 27.27 | 12.50 | 0.00 | 4.00 | 4.76 | 10.71 |

**Table 10.** Species and strains diversity in the sample.

| **Sample** | **MetaPhlan2** | **TAGMA** |
| --- | --- | --- |
| A1 | Alistipes_putredinis | Alistipes putredinis |
|  | Bacteroides_caccae  Bacteroides_dorei  Bacteroides_ovatus  Bacteroides_uniformis | Bacteroides dorei 5_1_36/D36  Bacteroides dorei CL02T12C06  Bacteroides fragilis HMW 615  Bacteroides fragilis str. 1007-1-F #8  Bacteroides ovatus 3_8_47FAA  Bacteroides ovatus ATCC 8483  Bacteroides ovatus str. 3725 D1 iv |
|  | Barnesiella_intestinihominis |  |
|  | Bifidobacterium_adolescentis  Bifidobacterium_bifidum | \| Bifidobacterium adolescentis \| \| --- \| \| Bifidobacterium adolescentis L2-32 \| \| Bifidobacterium bifidum \| \| Bifidobacterium bifidum ATCC 29521 = JCM 1255 = DSM 20456 \| \| Bifidobacterium breve \| \| Bifidobacterium breve DSM 20213 = JCM 1192 \| \| Bifidobacterium longum subsp. infantis ATCC 15697 = JCM 1222 = DSM 20088 \| \| Bifidobacterium longum subsp. longum ATCC 55813 \| \| Bifidobacterium longum subsp. longum F8 \| |
|  |  | Blautia obeum A2-162 |
|  |  | \| Clostridium acetireducens \| \| --- \| \| Clostridium disporicum \| \| Clostridium estertheticum \| \| Clostridium formicaceticum \| \| Clostridium perfringens \| |
|  |  | Coprococcus eutactus ATCC 27759 |
|  | Dialister_invisus | \| Dialister invisus \| \| --- \| \| Dialister invisus DSM 15470 \| |
|  |  | Dorea longicatena |
|  |  | Eubacterium ventriosum ATCC 27560 |
|  | Faecalibacterium_prausnitzii | \| Faecalibacterium prausnitzii A2-165 \| \| --- \| \| Faecalibacterium prausnitzii L2-6 \| \| Faecalibacterium prausnitzii M \| |
|  | Megamonas_hypermegale | Megamonas hypermegale ART12/1 |
|  | Parabacteroides_merdae | Parabacteroides merdae CL03T12C32 |
|  |  | \| Roseburia intestinalis L1-82 \| \| --- \| \| Roseburia inulinivorans \| |
|  | Prevotella_copri |  |
|  | Ruminococcus_bromii | Ruminococcus bromii L2-63 |
|  | Streptococcus_salivarius | \| Streptococcus pneumoniae \| \| --- \| \| Streptococcus salivarius 57.I \| \| Streptococcus salivarius CCHSS3 \| |
| A2 | Akkermansia_muciniphila | Akkermansia muciniphila ATCC BAA-835 |
|  | Alistipes_finegoldii  Alistipes_onderdonkii  Alistipes_putredinis | Alistipes putredinis |
|  | Bacteroides_caccae  Bacteroides_dorei  Bacteroides_ovatus  Bacteroides_thetaiotaomicron  Bacteroides_uniformis | \| Bacteroides dorei 5_1_36/D4 \| \| --- \| \| Bacteroides dorei CL02T12C06 \| \| Bacteroides ovatus 3_8_47FAA \| \| Bacteroides ovatus ATCC 8483 \| \| Bacteroides vulgatus str. 3975 RP4 \| |
|  | Barnesiella_intestinihominis |  |
|  | Bifidobacterium_bifidum  Bifidobacterium_longum  Bifidobacterium_pseudocatenulatum | Bifidobacterium bifidum ATCC 29521 = JCM 1255 = DSM 20456  Bifidobacterium bifidum |
|  |  | \| Blautia obeum \| \| --- \| \| Blautia obeum A2-162 \| |
|  |  | \| Clostridium colicanis \| \| --- \| \| Clostridium formicaceticum \| \| Clostridium hathewayi \| \| Clostridium ljungdahlii \| \| Clostridium neonatale \| \| Clostridium perfringens \| \| Clostridium perfringens WAL-14572 \| \| Clostridium sp. \| \| Clostridium tyrobutyricum \| |
|  |  | Coprococcus catus GD/7 |
|  | Dialister_invisus | Dialister invisus |
|  |  | Dorea longicatena |
|  | Eubacterium_rectale | \| Eubacterium ramulus ATCC 29099 \| \| --- \| \| Eubacterium rectale  Eubacterium ventriosum ATCC 27560 \| |
|  | Faecalibacterium_prausnitzii | \| Faecalibacterium prausnitzii A2-165 \| \| --- \| \| Faecalibacterium prausnitzii L2-6 \| \| Faecalibacterium prausnitzii M21/2 \| \| Faecalibacterium prausnitzii SL3/3 \| |
|  | Lachnospiraceae_bacterium |  |
|  |  | Megamonas hypermegale ART12/1 |
|  | Parabacteroides_merdae | \| Parabacteroides merdae ATCC 43184 \| \| --- \| \| Parabacteroides merdae CL03T12C32 \| |
|  | Prevotella_copri |  |
|  |  | \| Roseburia hominis \| \| --- \| \| Roseburia intestinalis M50/1 \| |
|  | Ruminococcus_bromii  Ruminococcus_callidus  Ruminococcus_obeum | \| Ruminococcus bicirculans \| \| --- \| \| Ruminococcus bromii L2-63 \| \| Ruminococcus callidus ATCC 27760 \| \| Ruminococcus gnavus ATCC 29149 \| \| Ruminococcus lactaris ATCC 29176 \| \| Ruminococcus torques L2-14 \| |
| A3 | Akkermansia_muciniphila |  |
|  | Bacteroides_dorei  Bacteroides_fragilis | \| Bacteroides dorei \| \| --- \| \| Bacteroides dorei 5_1_36/D4 \| \| Bacteroides dorei CL02T12C06 \| \| Bacteroides fragilis str. 1007-1-F #8 \| \| Bacteroides fragilis str. 3986 N(B)22 \| \| Bacteroides fragilis str. 3986T(B)10 \| \| Bacteroides ovatus 3_8_47FAA \| \| Bacteroides xylanisolvens \| \| Bacteroides xylanisolvens SD CC 1b \| |
|  |  | Blautia obeum |
|  |  | \| Butyrivibrio crossotus \| \| --- \| \| Butyrivibrio crossotus DSM 2876 \| |
|  |  | \| Clostridium botulinum \| \| --- \| \| Clostridium botulinum BKT015925 \| \| Clostridium nexile \| \| Clostridium perfringens \| \| Clostridium perfringens WAL-14572 \| \| Clostridium sporogenes \| |
|  |  | Dorea longicatena |
|  |  | \| Eggerthella lenta 1_1_60AFAA \| \| --- \| \| Eggerthella lenta DSM 2243 \| |
|  |  | Enterococcus faecium |
|  | Escherichia_coli  Escherichia_phage | \| Escherichia coli \| \| --- \| \| Escherichia coli CFT073 \| \| Escherichia coli G58-1 \| \| Escherichia coli UTI89 \| \| Escherichia fergusonii ATCC 35469 \| |
|  | Eubacterium_eligens  Eubacterium_rectale | \| Eubacterium eligens \| \| --- \| \| Eubacterium ramulus ATCC 29099 \| \| Eubacterium rectale \| \| Eubacterium rectale ATCC 33656 \| |
|  | Faecalibacterium_prausnitzii | \| Faecalibacterium prausnitzii \| \| --- \| \| Faecalibacterium prausnitzii L2-6 \| \| Faecalibacterium prausnitzii M21/2 \| |
|  | Lachnospiraceae_bacterium |  |
|  | Roseburia_intestinalis  Roseburia_inulinivorans | \| Roseburia hominis \| \| --- \| \| Roseburia intestinalis \| \| Roseburia intestinalis L1-82 \| \| Roseburia intestinalis M50/1 \| \| Roseburia intestinalis XB6B4 \| \| Roseburia inulinivorans \| \| Roseburia inulinivorans DSM 16841 \| |
|  |  | Ruminococcus bromii L2-63 |
| A4 | Akkermansia_muciniphila |  |
|  | Alistipes_finegoldii Alistipes_indistinctus Alistipes_putredinis Alistipes_shahii | Alistipes putredinis |
|  | Bacteroides_caccae Bacteroides_ovatus Bacteroides_thetaiotaomicron Bacteroides_vulgatus Bacteroides_xylanisolvens | \| Bacteroides cellulosilyticus \| \| --- \| \| Bacteroides ovatus str. 3725 D1 iv \| \| Bacteroides vulgatus \| \| Bacteroides vulgatus str. 3975 RP4 \| \| Bacteroides xylanisolvens SD CC 1b \| |
|  | Barnesiella_intestinihominis |  |
|  | Bifidobacterium_adolescentis | \| Bifidobacterium adolescentis \| \| --- \| \| Bifidobacterium adolescentis L2-32 \| \| Bifidobacterium stercoris JCM 15918 \| |
|  |  | \| Blautia obeum \| \| --- \| \| Blautia obeum A2-162 \| |
|  |  | \| Clostridium botulinum \| \| --- \| \| Clostridium formicaceticum \| \| Clostridium hathewayi \| \| Clostridium ljungdahlii \| \| Clostridium perfringens WAL-14572 \| \| Clostridium sporogenes \| |
|  | Coprococcus_eutactus | \| Coprococcus catus GD/7 \| \| --- \| \| Coprococcus eutactus ATCC 27759 \| |
|  |  | Desulfovibrio piger |
|  |  | Dorea longicatena |
|  |  | Enterococcus faecium 1,230,933 |
|  | Eubacterium_rectale | Eubacterium rectale ATCC 33656 |
|  | Faecalibacterium_prausnitzii | \| Faecalibacterium prausnitzii A2-165 \| \| --- \| \| Faecalibacterium prausnitzii L2-6 \| \| Faecalibacterium prausnitzii M21/2 \| \| Faecalibacterium prausnitzii SL3/3 \| |
|  | Lachnospiraceae_bacterium |  |
|  | Odoribacter_splanchnicus |  |
|  | Parabacteroides_merdae | \| Parabacteroides merdae \| \| --- \| \| Parabacteroides merdae CL03T12C32 \| |
|  | Prevotella_copri | Prevotella copri |
|  |  | \| Roseburia faecis \| \| --- \| \| Roseburia hominis \| \| Roseburia intestinalis \| \| Roseburia intestinalis L1-82 \| |
|  | Ruminococcus_bromii Ruminococcus_callidus Ruminococcus_lactaris Ruminococcus_torques | \| Ruminococcus bromii L2-63 1 \| \| --- \| \| Ruminococcus callidus ATCC 27760 \| \| Ruminococcus lactaris ATCC 29176 \| \| Ruminococcus torques L2-14 \| |
| A5 |  | Anaerotruncus colihominis DSM 17241 |
|  | Bacteroides_caccae Bacteroides_dorei Bacteroides_fragilis Bacteroides_ovatus Bacteroides_plebeius Bacteroides_uniformis Bacteroides_vulgatus | \| Bacteroides dorei \| \| --- \| \| Bacteroides fragilis HMW 615 \| \| Bacteroides fragilis str. 1007-1-F #8 \| \| Bacteroides ovatus 3_8_47FAA \| \| Bacteroides ovatus ATCC 8483 \| \| Bacteroides vulgatus \| \| Bacteroides vulgatus str. 3975 RP4 \| \| Bacteroides xylanisolvens \| |
|  |  | Blautia obeum |
|  | Barnesiella_intestinihominis |  |
|  |  | \| Clostridium botulinum \| \| --- \| \| Clostridium nexile \| \| Clostridium perfringens WAL-14572 \| \| Clostridium sartagoforme AAU1 \| |
|  | Dialister_invisus | \| Dialister invisus \| \| --- \| \| Dialister invisus DSM 15470 \| |
|  |  | Dorea longicatena |
|  |  | Enterococcus faecium 1,230,933 |
|  | Eubacterium_eligens Eubacterium_rectale | \| Eubacterium eligens \| \| --- \| \| Eubacterium rectale ATCC 33656 \| \| Eubacterium siraeum \| |
|  | Faecalibacterium_prausnitzii | \| Faecalibacterium prausnitzii \| \| --- \| \| Faecalibacterium prausnitzii A2-165 \| \| Faecalibacterium prausnitzii L2-6 \| \| Faecalibacterium prausnitzii M21/2 \| \| Faecalibacterium prausnitzii SL3/3 \| |
|  | Lachnospiraceae_bacterium |  |
|  | Odoribacter_splanchnicus |  |
|  | Parabacteroides_merdae | \| Parabacteroides johnsonii \| \| --- \| \| Parabacteroides merdae CL03T12C32 \| |
|  | Roseburia_hominis | \| Roseburia hominis \| \| --- \| \| Roseburia intestinalis L1-82 \| |
|  | Ruminococcus_torques | Ruminococcus bicirculans  Ruminococcus torques L2-14 |
|  |  | \| Streptococcus thermophilus \| \| --- \| \| Streptococcus thermophilus TH982 \| |
| A6 | Akkermansia_muciniphila |  |
|  | Alistipes_putredinis |  |
|  | Bacteroides_caccae Bacteroides_fragilis Bacteroides_vulgatus | \| Bacteroides clarus YIT 12056 \| \| --- \| \| Bacteroides fragilis str. 1007-1-F #8 \| \| Bacteroides fragilis str. 3783N1-2 \| \| Bacteroides fragilis str. 3986 N(B)22 \| \| Bacteroides fragilis str. 3996 N(B) 6 \| \| Bacteroides ovatus 3_8_47FAA \| \| Bacteroides vulgatus \| \| Bacteroides vulgatus PC510 \| \| Bacteroides vulgatus str. 3975 RP4 \| \| Bacteroides xylanisolvens \| |
|  | Bifidobacterium_adolescentis Bifidobacterium_bifidum Bifidobacterium_longum | \| Bifidobacterium adolescentis \| \| --- \| \| Bifidobacterium adolescentis L2-32 \| \| Bifidobacterium bifidum \| \| Bifidobacterium longum subsp. infantis 157F \| \| Bifidobacterium longum subsp. longum ATCC 55813 \| \| Bifidobacterium longum subsp. longum F8 \| \| Bifidobacterium stercoris JCM 15918 \| |
|  |  | \| Blautia obeum \| \| --- \| \| Blautia obeum A2-162 \| \| Blautia obeum ATCC 29174 \| |
|  | Catenibacterium_mitsuokai | Catenibacterium sp. |
|  | Collinsella_aerofaciens | Clostridium perfringens WAL-14572 |
|  |  | Coprococcus comes ATCC 27758 |
|  |  | \| Dorea formicigenerans ATCC 27755 \| \| --- \| \| Dorea longicatena \| |
|  | Escherichia_coli | \| Escherichia coli G58-1 \| \| --- \| \| Escherichia coli IS5 \| \| Escherichia coli MS 21-1 \| \| Escherichia coli O26:H11 str. 11368 \| \| Escherichia coli PA9 \| |
|  | Eubacterium_biforme | Eubacterium sp. |
|  | Faecalibacterium_prausnitzii | \| Faecalibacterium prausnitzii \| \| --- \| \| Faecalibacterium prausnitzii A2-165 \| \| Faecalibacterium prausnitzii L2-6 1 \| \| Faecalibacterium prausnitzii M21/2 \| \| Faecalibacterium prausnitzii SL3/3 \| |
|  |  | Lactobacillus ruminis |
|  | Megasphaera_elsdenii | Megamonas hypermegale ART12/1 |
|  | Parabacteroides_merdae | \| Parabacteroides merdae \| \| --- \| \| Parabacteroides merdae ATCC 43184 \| \| Parabacteroides merdae CL03T12C32 \| |
|  | Prevotella_copri | Prevotella copri |
|  |  | \| Roseburia faecis \| \| --- \| \| Roseburia hominis \| \| Roseburia intestinalis \| \| Roseburia inulinivorans DSM 16841 \| |
|  | Ruminococcus_bromii Ruminococcus_obeum | \| Ruminococcus bromii L2-63 \| \| --- \| \| Ruminococcus lactaris ATCC 29176 \| |
| A7 | Alistipes_finegoldii Alistipes_indistinctus Alistipes_onderdonkii Alistipes_putredinis Alistipes_shahii | Alistipes putredinis |
|  | Bacteroides_caccae Bacteroides_fragilis Bacteroides_uniformis Bacteroides_vulgatus | \| Bacteroides faecis \| \| --- \| \| Bacteroides fragilis str. 1007-1-F #8 \| \| Bacteroides fragilis str. 3783N1-2 \| \| Bacteroides fragilis str. 3986 N(B)22 \| \| Bacteroides fragilis str. 3996 N(B) 6 \| \| Bacteroides ovatus 3_8_47FAA \| \| Bacteroides sp. \| \| Bacteroides stercoris \| \| Bacteroides vulgatus \| \| Bacteroides vulgatus PC510 \| \| Bacteroides vulgatus str. 3975 RP4 \| \| Bacteroides xylanisolvens \| \| Bacteroides xylanisolvens SD CC 1b \| |
|  | Barnesiella_intestinihominis |  |
|  | Bifidobacterium_adolescentis Bifidobacterium_bifidum Bifidobacterium_longum | \| Bifidobacterium adolescentis \| \| --- \| \| Bifidobacterium adolescentis L2-32 \| \| Bifidobacterium bifidum \| \| Bifidobacterium longum subsp. infantis 157F \| \| Bifidobacterium longum subsp. longum ATCC 55813 \| \| Bifidobacterium longum subsp. longum F8 \| \| Bifidobacterium stercoris JCM 15918 \| |
|  |  | \| Blautia obeum \| \| --- \| \| Blautia obeum A2-162 \| |
|  | Catenibacterium_mitsuokai | Catenibacterium sp. |
|  |  | Clostridium perfringens WAL-14572 |
|  | Collinsella_aerofaciens | Collinsella sp. |
|  | Coprococcus_comes | \| Coprococcus catus GD/7 \| \| --- \| \| Coprococcus comes ATCC 27758 \| |
|  |  | Dorea longicatena |
|  |  | Escherichia coli G58-1 |
|  | Eubacterium_rectale | \| Eubacterium rectale \| \| --- \| \| Eubacterium rectale ATCC 33656 \| |
|  | Faecalibacterium_prausnitzii | \| Faecalibacterium prausnitzii \| \| --- \| \| Faecalibacterium prausnitzii A2-165 \| \| Faecalibacterium prausnitzii L2-6 1 \| \| Faecalibacterium prausnitzii M21/2 \| \| Faecalibacterium prausnitzii SL3/3 \| |
|  | Lactobacillus_ruminis | Lactobacillus ruminis |
|  | Megasphaera_elsdenii |  |
|  | Parabacteroides_merdae | \| Parabacteroides merdae ATCC 43184 \| \| --- \| \| Parabacteroides merdae CL03T12C32 \| |
|  | Prevotella_copri | Prevotella copri |
|  |  | \| Roseburia hominis \| \| --- \| \| Roseburia intestinalis M50/1 \| |
|  | Ruminococcus_callidus | \| Ruminococcus bromii L2-63 \| \| --- \| \| Ruminococcus callidus ATCC 27760 \| \| Ruminococcus lactaris ATCC 29176 \| |
| A8 | Alistipes_finegoldii Alistipes_putredinis Alistipes_shahii | Alistipes putredinis |
|  | Bacteroides_caccae Bacteroides_eggerthii Bacteroides_ovatus Bacteroides_stercoris Bacteroides_uniformis Bacteroides_vulgatus | \| Bacteroides intestinalis \| \| --- \| \| Bacteroides ovatus ATCC 8483 \| \| Bacteroides sp. \| \| Bacteroides stercoris \| \| Bacteroides vulgatus \| \| Bacteroides vulgatus str. 3775 SR(B) 19 \| \| Bacteroides vulgatus str. 3975 RP4 \| |
|  | Bifidobacterium_adolescentis | \| Bifidobacterium adolescentis \| \| --- \| \| Bifidobacterium adolescentis L2-32 \| \| Bifidobacterium stercoris JCM 15918 \| |
|  |  | Blautia obeum A2-162 |
|  |  | Butyrivibrio crossotus |
|  |  | \| Clostridium formicaceticum \| \| --- \| \| Clostridium hathewayi \| \| Clostridium homopropionicum \| \| Clostridium ljungdahlii \| \| Clostridium perfringens WAL-14572 \| |
|  | Coprococcus_sp | Coprococcus comes ATCC 27758 |
|  | Dialister_invisus | \| Dialister invisus \| \| --- \| \| Dialister invisus DSM 15470 \| |
|  |  | Dorea longicatena |
|  | Eubacterium_eligens Eubacterium_rectale | \| Eubacterium eligens \| \| --- \| \| Eubacterium rectale \| \| Eubacterium rectale ATCC 33656 \| |
|  | Faecalibacterium_prausnitzii | \| Faecalibacterium prausnitzii \| \| --- \| \| Faecalibacterium prausnitzii A2-165 \| \| Faecalibacterium prausnitzii L2-6 2 \| \| Faecalibacterium prausnitzii M21/2 \| \| Faecalibacterium prausnitzii SL3/3 \| |
|  |  | Odoribacter splanchnicus |
|  |  | Parabacteroides merdae CL03T12C32 |
|  | Roseburia_intestinalis | \| Roseburia hominis \| \| --- \| \| Roseburia intestinalis L1-82 \| \| Roseburia intestinalis M50/1 \| \| Roseburia inulinivorans \| \| Roseburia inulinivorans DSM 16841 \| |
|  |  | \| Ruminococcus bromii L2-63 \| \| --- \| \| Ruminococcus lactaris ATCC 29176 \| |
| A9 | Bacteroides_fragilis Bacteroides_ovatus | \| Bacteroides fragilis HMW 615 \| \| --- \| \| Bacteroides fragilis str. 1007-1-F #8 \| \| Bacteroides fragilis str. 3986T(B)10 \| \| Bacteroides ovatus 3_8_47FAA \| \| Bacteroides sp. \| \| Bacteroides xylanisolvens \| \| Bacteroides xylanisolvens SD CC 1b \| \| Bacteroides xylanisolvens XB1A \| |
|  |  | \| Blautia obeum \| \| --- \| \| Blautia obeum ATCC 29174 \| |
|  | Bifidobacterium_longum Bifidobacterium_pseudocatenulatum |  |
|  |  | \| Clostridium botulinum \| \| --- \| \| Clostridium formicaceticum \| \| Clostridium phage c-st \| |
|  |  | Coprococcus comes ATCC 27758 |
|  | Dialister_invisus | Dialister invisus DSM 15470 |
|  | Dorea_longicatena | \| Dorea formicigenerans ATCC 27755 \| \| --- \| \| Dorea longicatena \| |
|  | Escherichia_coli | \| Escherichia coli ATCC 8739 \| \| --- \| \| Escherichia coli O32:H37 str. P4 \| |
|  | Eubacterium_hallii | Eubacterium eligens |
|  | Faecalibacterium_prausnitzii | \| Faecalibacterium prausnitzii A2-165 \| \| --- \| \| Faecalibacterium prausnitzii L2-6 2 \| \| Faecalibacterium prausnitzii M21/2 \| |
|  | Haemophilus_parainfluenzae |  |
|  | Lachnospiraceae_bacterium |  |
|  | Roseburia_intestinalis Roseburia_inulinivorans | \| Roseburia hominis \| \| --- \| \| Roseburia intestinalis \| \| Roseburia intestinalis L1-82 \| \| Roseburia intestinalis XB6B4 \| \| Roseburia inulinivorans \| \| Roseburia inulinivorans DSM 16841 \| |
|  | Ruminococcus_lactaris Ruminococcus_torques | \| Ruminococcus bromii L2-63 \| \| --- \| \| Ruminococcus lactaris ATCC 29176 \| \| Ruminococcus torques L2-14 \| |
|  | Streptococcus_parasanguinis Streptococcus_salivarius | Streptococcus parasanguinis F0449 |
| A10 | Acidaminococcus_fermentans | \| Acidaminococcus fermentans \| \| --- \| \| Acidaminococcus fermentans DSM 20731 \| |
|  | Bacteroides_vulgatus | \| Bacteroides fragilis str. 3783N1-2 \| \| --- \| \| Bacteroides fragilis str. 3996 N(B) \| \| Bacteroides ovatus str. 3725 D1 iv \| \| Bacteroides sp. \| \| Bacteroides vulgatus \| \| Bacteroides vulgatus PC510 \| \| Bacteroides vulgatus str. 3975 RP4 \| |
|  | Bifidobacterium_adolescentis Bifidobacterium_bifidum | \| Bifidobacterium adolescentis \| \| --- \| \| Bifidobacterium adolescentis L2-32 \| \| Bifidobacterium animalis subsp. lactis BB-12 \| \| Bifidobacterium bifidum \| \| Bifidobacterium longum subsp. longum F8 \| \| Bifidobacterium stercoris JCM 15918 \| \| Bifidobacterium thermacidophilum subsp. porcinum \| |
|  |  | \| Blautia obeum A2-162 \| \| --- \| \| Blautia obeum ATCC 29174 \| |
|  | Catenibacterium_mitsuokai | Catenibacterium sp. |
|  |  | \| Clostridium formicaceticum \| \| --- \| \| Clostridium perfringens \| \| Clostridium perfringens WAL-14572 \| |
|  | Collinsella_aerofaciens | Collinsella sp. |
|  |  | \| Coprococcus catus GD/7 \| \| --- \| \| Coprococcus eutactus ATCC 27759 \| |
|  | Dorea_longicatena | \| Dorea formicigenerans 4_6_53AFAA \| \| --- \| \| Dorea formicigenerans ATCC 27755 \| \| Dorea longicatena \| |
|  |  | Enterococcus faecium 1,230,933 |
|  |  | \| Escherichia coli G58-1 \| \| --- \| \| Escherichia coli MS 21-1 \| \| Escherichia coli PA9 \| |
|  | Eubacterium_biforme Eubacterium_rectale | \| Eubacterium rectale \| \| --- \| \| Eubacterium rectale ATCC 33656 \| |
|  | Faecalibacterium_prausnitzii | \| Faecalibacterium prausnitzii \| \| --- \| \| Faecalibacterium prausnitzii A2-165 \| \| Faecalibacterium prausnitzii L2-6 1 \| \| Faecalibacterium prausnitzii M21/2 \| \| Faecalibacterium prausnitzii SL3/3 \| |
|  |  | \| Lactobacillus acidophilus ATCC 4796 \| \| --- \| \| Lactobacillus ruminis \| |
|  | Megasphaera_elsdenii | Megasphaera elsdenii |
|  | Mitsuokella_multacida | Mitsuokella multacida DSM 20544 |
|  | Parabacteroides_merdae | \| Parabacteroides merdae \| \| --- \| \| Parabacteroides merdae CL03T12C32 \| |
|  | Phascolarctobacterium_succinatutens |  |
|  | Prevotella_copri | Prevotella copri |
|  | Roseburia_inulinivorans | \| Roseburia faecis \| \| --- \| \| Roseburia hominis \| \| Roseburia intestinalis \| \| Roseburia intestinalis M50/1 \| \| Roseburia intestinalis XB6B4 \| \| Roseburia inulinivorans \| \| Roseburia inulinivorans DSM 16841 \| |
|  | Ruminococcus_sp | \| Ruminococcus bromii L2-63 \| \| --- \| \| Ruminococcus lactaris ATCC 29176 \| \| Ruminococcus obeum \| \| Ruminococcus sp. \| \| Ruminococcus torques ATCC 27756 \| \| Ruminococcus torques L2-14 \| |
|  | Streptococcus_thermophilus | \| Streptococcus thermophilus M17PTZA496 \| \| --- \| \| Streptococcus thermophilus TH982 \| |
| A11 | Bacteroides_eggerthii Bacteroides_massiliensis Bacteroides_ovatus Bacteroides_uniformis Bacteroides_vulgatus | \| Bacteroides ovatus str. 3725 D1 iv \| \| --- \| \| Bacteroides vulgatus \| \| Bacteroides vulgatus PC510 \| \| Bacteroides vulgatus str. 3975 RP4 \| |
|  |  | \| Blautia hydrogenotrophica DSM 10507 \| \| --- \| \| Blautia obeum \| \| Blautia obeum A2-162 \| |
|  | Catenibacterium_mitsuokai | Catenibacterium sp. |
|  | Clostridium_bolteae | \| Clostridium formicaceticum \| \| --- \| \| Clostridium perfringens WAL-14572 \| |
|  | Collinsella_aerofaciens | Collinsella sp. |
|  | Coprococcus_comes | \| Coprococcus comes \| \| --- \| \| Coprococcus comes ATCC 27758 \| |
|  | Dorea_longicatena | Dorea longicatena |
|  |  | Enterococcus faecium 1,230,933 |
|  | Eubacterium_rectale | \| Eubacterium rectale \| \| --- \| \| Eubacterium rectale ATCC 33656 \| |
|  | Faecalibacterium_prausnitzii | \| Faecalibacterium prausnitzii \| \| --- \| \| Faecalibacterium prausnitzii A2-165 \| \| Faecalibacterium prausnitzii L2-6 \| \| Faecalibacterium prausnitzii M21/2 \| |
|  | Lachnospiraceae_bacterium |  |
|  | Lactobacillus_sakei | \| Lactobacillus sakei subsp. carnosus DSM 15831 \| \| --- \| \| Lactobacillus sakei subsp. sakei 23K \| |
|  | Lactococcus_lactis |  |
|  | Parabacteroides_merdae | Parabacteroides merdae |
|  | Roseburia_intestinalis Roseburia_inulinivorans | \| Roseburia faecis \| \| --- \| \| Roseburia hominis \| \| Roseburia intestinalis \| \| Roseburia intestinalis L1-82 \| \| Roseburia intestinalis M50/1 \| \| Roseburia intestinalis XB6B4 \| \| Roseburia inulinivorans \| \| Roseburia inulinivorans DSM 16841 \| |
|  | Ruminococcus_bromii Ruminococcus_lactaris Ruminococcus_obeum Ruminococcus_torques | \| Ruminococcus bromii L2-63 \| \| --- \| \| Ruminococcus gnavus ATCC 29149 \| \| Ruminococcus lactaris ATCC 29176 \| \| Ruminococcus torques L2-14 \| |
| A12 | Alistipes_putredinis | Alistipes sp. |
|  | Bacteroides_caccae Bacteroides_thetaiotaomicron Bacteroides_vulgatus | \| Bacteroides faecis \| \| --- \| \| Bacteroides vulgatus \| \| Bacteroides vulgatus str. 3975 RP4 \| \| Bacteroides xylanisolvens \| |
|  | Bifidobacterium_adolescentis | \| Bifidobacterium adolescentis \| \| --- \| \| Bifidobacterium adolescentis L2-32 \| \| Bifidobacterium stercoris JCM 15918 \| |
|  |  | \| Blautia obeum \| \| --- \| \| Blautia obeum ATCC 29174 \| |
|  |  | \| Clostridium asparagiforme DSM 15981 \| \| --- \| \| Clostridium botulinum \| \| Clostridium botulinum F \| \| Clostridium perfringens \| \| Clostridium perfringens WAL-14572 \| \| Clostridium sp. \| \| Clostridium sporogenes \| \| Clostridium tetanomorphum DSM 665 \| |
|  | Collinsella_aerofaciens | Collinsella sp. |
|  | Coprococcus_comes | Coprococcus comes ATCC 27758 |
|  |  | Desulfovibrio piger |
|  |  | \| Dorea formicigenerans 4_6_53AFAA \| \| --- \| \| Dorea formicigenerans ATCC 27755 \| \| Dorea longicatena \| |
|  |  | Enterococcus faecium 1,230,933 |
|  | Eubacterium_biforme Eubacterium_eligens Eubacterium_rectale | \| Eubacterium eligens \| \| --- \| \| Eubacterium ramulus ATCC 29099 \| \| Eubacterium rectale \| \| Eubacterium rectale ATCC 33656 \| |
|  | Faecalibacterium_prausnitzii | \| Faecalibacterium prausnitzii A2-165 \| \| --- \| \| Faecalibacterium prausnitzii L2-6 \| \| Faecalibacterium prausnitzii M21/2 \| |
|  | Lachnospiraceae_bacterium |  |
|  | Prevotella_copri | Prevotella copri |
|  |  | \| Roseburia intestinalis \| \| --- \| \| Roseburia intestinalis L1-82 \| \| Roseburia inulinivorans \| |
|  | Ruminococcus_lactaris Ruminococcus_obeum Ruminococcus_torques | \| Ruminococcus bicirculans \| \| --- \| \| Ruminococcus lactaris ATCC 29176 \| \| Ruminococcus sp. \| \| Ruminococcus torques L2-14 \| |
| A13 | Bacteroides_plebeius | \| Bacteroides vulgatus str. 3775 SR(B) 19 \| \| --- \| \| Bacteroides xylanisolvens \| |
|  |  | \| Blautia obeum \| \| --- \| \| Blautia obeum A2-162 \| |
|  | Bifidobacterium_longum |  |
|  | Catenibacterium_mitsuokai | Catenibacterium sp. |
|  | Clostridium_bartlettii | Clostridium sp. |
|  |  | Dorea longicatena |
|  | Enterococcus_faecium | Enterococcus faecium EnGen0178 |
|  | Escherichia_coli | \| Escherichia coli \| \| --- \| \| Escherichia coli CFT073 \| \| Escherichia coli G58-1 \| \| Escherichia coli IS5 \| \| Escherichia coli UTI89 \| |
|  | Faecalibacterium_prausnitzii | \| Faecalibacterium prausnitzii L2-6 \| \| --- \| \| Faecalibacterium prausnitzii M21/2 \| |
|  | Prevotella_copri | Prevotella sp. |
|  |  | Roseburia hominis |
|  | Ruminococcus_sp |  |
| A14 | Akkermansia_muciniphila | Anaerotruncus colihominis DSM 17241 |
|  | Alistipes_putredinis |  |
|  | Bacteroides_caccae Bacteroides_dorei Bacteroides_fragilis Bacteroides_uniformis Bacteroides_xylanisolvens | \| Bacteroides caccae ATCC 43185 \| \| --- \| \| Bacteroides coprocola DSM 17136 \| \| Bacteroides dorei \| \| Bacteroides dorei 5_1_36/D4 \| \| Bacteroides dorei CL02T12C06 \| \| Bacteroides fragilis CL03T00C08 \| \| Bacteroides fragilis str. 1007-1-F #8 \| \| Bacteroides fragilis str. 3986 N(B)22 \| \| Bacteroides fragilis str. 3986T(B)10 \| \| Bacteroides fragilis str. 3988 T1 \| \| Bacteroides sp. \| \| Bacteroides xylanisolvens SD CC 1b \| |
|  |  |  |
|  | Bifidobacterium_adolescentis Bifidobacterium_longum | \| Bifidobacterium adolescentis \| \| --- \| \| Bifidobacterium adolescentis L2-32 \| \| Bifidobacterium longum subsp. longum ATCC 55813 \| |
|  |  | \| Blautia obeum \| \| --- \| \| Blautia obeum A2-162 \| \| Blautia obeum ATCC 29174 \| |
|  | Clostridium_leptum | \| Clostridium botulinum \| \| --- \| \| Clostridium coskatii \| \| Clostridium disporicum \| \| Clostridium estertheticum \| \| Clostridium formicaceticum \| \| Clostridium hathewayi \| \| Clostridium ljungdahlii \| \| Clostridium perfringens \| \| Clostridium perfringens WAL-14572 \| \| Clostridium sporogenes \| |
|  |  | \| Coprococcus catus GD/7 \| \| --- \| \| Coprococcus eutactus ATCC 27759 \| |
|  |  | \| Dorea formicigenerans 4_6_53AFAA \| \| --- \| \| Dorea formicigenerans ATCC 27755 \| \| Dorea longicatena \| |
|  |  | Eggerthella lenta DSM 2243 |
|  |  | \| Enterococcus faecium \| \| --- \| \| Enterococcus faecium 1,230,933 \| |
|  |  | \| Escherichia coli ATCC 8739 \| \| --- \| \| Escherichia coli DORA_A_5_14_21 \| \| Escherichia coli G58-1 \| \| Escherichia coli O32:H37 str. P4 \| |
|  | Eubacterium_biforme Eubacterium_rectale | \| Eubacterium ramulus ATCC 29099 \| \| --- \| \| Eubacterium rectale \| \| Eubacterium rectale ATCC 33656 \| |
|  | Faecalibacterium_prausnitzii | \| Faecalibacterium prausnitzii A2-165 \| \| --- \| \| Faecalibacterium prausnitzii L2-6 \| \| Faecalibacterium prausnitzii M21/2 \| \| Faecalibacterium prausnitzii SL3/3 \| |
|  |  | Gordonibacter pamelaeae 7-10-1-b |
|  | Lachnospiraceae_bacterium |  |
|  | Roseburia_hominis | \| Roseburia hominis \| \| --- \| \| Roseburia intestinalis L1-82 \| \| Roseburia intestinalis M50/1 \| \| Roseburia intestinalis XB6B4 \| \| Roseburia inulinivorans \| |
|  | Ruminococcus_bromii Ruminococcus_gnavus Ruminococcus_obeum Ruminococcus_torques | \| Ruminococcus bicirculans \| \| --- \| \| Ruminococcus bromii L2-63 \| \| Ruminococcus gnavus ATCC 29149 \| \| Ruminococcus lactaris ATCC 29176 \| \| Ruminococcus torques ATCC 27756 \| \| Ruminococcus torques L2-14 \| |
| A15 | Anaerostipes_hadrus | \| Anaerostipes hadrus \| \| --- \| \| Anaerostipes hadrus DSM 3319 \| |
|  | Bacteroides_dorei Bacteroides_ovatus Bacteroides_thetaiotaomicron Bacteroides_uniformis | \| Bacteroides coprocola DSM 17136 \| \| --- \| \| Bacteroides dorei 5_1_36/D4 \| \| Bacteroides faecis \| \| Bacteroides ovatus 3_8_47FAA \| \| Bacteroides ovatus ATCC 8483 \| \| Bacteroides xylanisolvens \| |
|  | Bifidobacterium_adolescentis Bifidobacterium_breve Bifidobacterium_longum | \| Bifidobacterium adolescentis \| \| --- \| \| Bifidobacterium adolescentis L2-32 \| \| Bifidobacterium breve \| \| Bifidobacterium breve DSM 20213 = JCM 1192 \| \| Bifidobacterium longum subsp. infantis ATCC 15697 = JCM 1222 = DSM 20088 \| \| Bifidobacterium longum subsp. infantis CCUG 52486 \| \| Bifidobacterium longum subsp. longum 1-6B \| \| Bifidobacterium longum subsp. longum ATCC 55813 \| \| Bifidobacterium longum subsp. longum F8 \| \| Bifidobacterium stercoris JCM 15918 \| \| Bifidobacterium thermacidophilum subsp. porcinum \| |
|  |  | \| Blautia obeum \| \| --- \| \| Blautia obeum A2-162 \| |
|  |  | Clostridium baratii |
|  | Collinsella_aerofaciens | Collinsella sp. |
|  |  | \| Dorea formicigenerans ATCC 27755 \| \| --- \| \| Dorea longicatena \| |
|  |  | Eggerthella lenta DSM 2243 |
|  |  | \| Enterococcus faecium \| \| --- \| \| Enterococcus faecium 1,230,933 \| |
|  | Escherichia_coli | \| Escherichia coli \| \| --- \| \| Escherichia coli ATCC 8739 \| \| Escherichia coli DORA_A_5_14_21 \| \| Escherichia coli G58-1 \| \| Escherichia coli O26:H11 str. 11368 \| \| Escherichia coli O32:H37 str. P4 \| |
|  | Eubacterium_hallii Eubacterium_rectale | Eubacterium rectale ATCC 33656 |
|  | Faecalibacterium_prausnitzii | \| Faecalibacterium prausnitzii L2-6 \| \| --- \| \| Faecalibacterium prausnitzii M21/2 \| |
|  | Lachnospiraceae_bacterium |  |
|  |  | Lactobacillus rogosae |
|  | Parasutterella_excrementihominis |  |
|  |  | \| Roseburia hominis \| \| --- \| \| Roseburia intestinalis \| \| Roseburia intestinalis M50/1 \| \| Roseburia intestinalis XB6B4 \| |
|  |  | \| Ruminococcus bromii L2-63 \| \| --- \| \| Ruminococcus torques ATCC 27756 \| \| Ruminococcus torques L2-14 \| |
| B1 | Alistipes_onderdonkii |  |
|  |  | Anaerostipes hadrus DSM 3319 |
|  | Bacteroides_dorei Bacteroides_ovatus Bacteroides_uniformis Bacteroides_vulgatus | \| Bacteroides dorei CL02T12C06 \| \| --- \| \| Bacteroides ovatus 3_8_47FAA \| \| Bacteroides ovatus ATCC 8483 \| \| Bacteroides stercoris \| \| Bacteroides vulgatus \| \| Bacteroides vulgatus PC510 \| \| Bacteroides vulgatus str. 3775 SR(B) \| \| Bacteroides vulgatus str. 3975 RP4 \| \| Bacteroides xylanisolvens \| |
|  | Bifidobacterium_longum |  |
|  |  | \| Blautia obeum \| \| --- \| \| Blautia obeum A2-162 \| |
|  |  | \| Clostridium asparagiforme DSM 15981 \| \| --- \| \| Clostridium botulinum \| \| Clostridium clostridioforme \| \| Clostridium perfringens WAL-14572 \| |
|  | Dialister_invisus | \| Dialister invisus \| \| --- \| \| Dialister invisus DSM 15470 \| |
|  |  | Dorea longicatena |
|  |  | Enterococcus faecium 1,230,933 |
|  | Eubacterium_eligens Eubacterium_rectale Eubacterium_ventriosum | \| Eubacterium eligens \| \| --- \| \| Eubacterium rectale \| \| Eubacterium rectale ATCC 33656 \| \| Eubacterium ventriosum ATCC 27560 \| |
|  | Faecalibacterium_prausnitzii | \| Faecalibacterium prausnitzii \| \| --- \| \| Faecalibacterium prausnitzii A2-165 \| \| Faecalibacterium prausnitzii L2-6 \| \| Faecalibacterium prausnitzii M21/2 \| \| Faecalibacterium prausnitzii SL3/3 \| |
|  | Haemophilus_parainfluenzae |  |
|  |  | \| Lactobacillus casei W56 \| \| --- \| \| Lactobacillus paracasei subsp. paracasei CNCM I-4648 \| \| Lactobacillus paracasei subsp. paracasei Lpp126 \| \| Lactobacillus paracasei subsp. paracasei Lpp71 \| |
|  | Roseburia_inulinivorans | \| Roseburia faecis \| \| --- \| \| Roseburia hominis \| \| Roseburia intestinalis M50/1 \| \| Roseburia intestinalis XB6B4 \| \| Roseburia inulinivorans \| \| Roseburia inulinivorans DSM 16841 \| |
|  | Ruminococcus_bromii Ruminococcus_torques | \| Ruminococcus bromii L2-63 \| \| --- \| \| Ruminococcus callidus ATCC 27760 \| \| Ruminococcus torques L2-14 \| |
|  |  | Veillonella atypica ACS-134-V-Col7a |
| B2 | Alistipes_onderdonkii |  |
|  | Bacteroides_dorei Bacteroides_ovatus Bacteroides_uniformis Bacteroides_vulgatus | \| Bacteroides dorei CL02T12C06 \| \| --- \| \| Bacteroides ovatus 3_8_47FAA \| \| Bacteroides ovatus ATCC 8483 \| \| Bacteroides stercoris \| \| Bacteroides vulgatus \| \| Bacteroides vulgatus PC510 \| \| Bacteroides vulgatus str. 3975 RP4 \| \| Bacteroides xylanisolvens \| |
|  |  | \| Blautia obeum \| \| --- \| \| Blautia obeum A2-162 \| |
|  | Bifidobacterium_longum |  |
|  |  | \| Clostridium botulinum \| \| --- \| \| Clostridium perfringens WAL-14572 \| |
|  | Dialister_invisus | \| Dialister invisus \| \| --- \| \| Dialister invisus DSM 15470 \| |
|  |  | Dorea longicatena |
|  |  | Enterococcus faecium 1,230,933 |
|  | Eubacterium_eligens Eubacterium_rectale Eubacterium_ventriosum | \| Eubacterium rectale \| \| --- \| \| Eubacterium rectale ATCC 33656 \| \| Eubacterium sp. \| \| Eubacterium ventriosum ATCC 27560 \| |
|  | Faecalibacterium_prausnitzii | \| Faecalibacterium prausnitzii \| \| --- \| \| Faecalibacterium prausnitzii A2-165 \| \| Faecalibacterium prausnitzii L2-6 \| \| Faecalibacterium prausnitzii M21/2 \| \| Faecalibacterium prausnitzii SL3/3 \| |
|  | Roseburia_inulinivorans | \| Roseburia intestinalis XB6B4 \| \| --- \| \| Roseburia inulinivorans \| |
|  | Ruminococcus_bromii | Ruminococcus bromii L2-63 1 |
| B3 | Bacteroides_dorei Bacteroides_fragilis Bacteroides_ovatus Bacteroides_uniformis | \| Bacteroides dorei \| \| --- \| \| Bacteroides dorei 5_1_36/D4 \| \| Bacteroides fragilis str. 1007-1-F #8 \| \| Bacteroides fragilis str. 3986 N(B)22 \| \| Bacteroides fragilis str. 3986T(B)10 \| \| Bacteroides fragilis str. Ds-233 \| |
|  | Bifidobacterium_adolescentis Bifidobacterium_longum | \| Bifidobacterium adolescentis \| \| --- \| \| Bifidobacterium animalis subsp. lactis BB-12 \| \| Bifidobacterium longum subsp. longum 1-6B \| |
|  |  | \| Blautia obeum \| \| --- \| \| Blautia obeum A2-162 \| |
|  |  | Clostridium perfringens WAL-14572 |
|  | Collinsella_aerofaciens | Collinsella sp. |
|  |  | \| Coprococcus catus GD/7 \| \| --- \| \| Coprococcus comes ATCC 27758 \| |
|  | Dialister_invisus | Dialister invisus |
|  |  | Dorea longicatena |
|  |  | Enterococcus faecium 1,230,933 |
|  | Escherichia_coli | \| Escherichia coli G58-1 \| \| --- \| \| Escherichia coli UTI89 \| |
|  |  | \| Eubacterium eligens \| \| --- \| \| Eubacterium ventriosum ATCC 27560 \| |
|  | Faecalibacterium_prausnitzii | \| Faecalibacterium prausnitzii A2-165 \| \| --- \| \| Faecalibacterium prausnitzii L2-6 \| \| Faecalibacterium prausnitzii M21/2 \| \| Faecalibacterium prausnitzii SL3/3 \| |
|  | Parabacteroides_merdae | \| Parabacteroides merdae \| \| --- \| \| Parabacteroides merdae CL03T12C32 \| |
|  | Roseburia_hominis Roseburia_inulinivorans | \| Roseburia faecis \| \| --- \| \| Roseburia hominis \| \| Roseburia intestinalis \| \| Roseburia intestinalis M50/1 \| \| Roseburia inulinivorans \| \| Roseburia inulinivorans DSM 16841 \| |
|  | Ruminococcus_lactaris Ruminococcus_torques | \| Ruminococcus bromii L2-63 \| \| --- \| \| Ruminococcus lactaris ATCC 29176 \| \| Ruminococcus sp. \| \| Ruminococcus torques ATCC 27756 \| \| Ruminococcus torques L2-14 \| |
| B4 | Akkermansia_muciniphila |  |
|  | Alistipes_finegoldii |  |
|  | Anaerostipes_hadrus | \| Anaerostipes hadrus \| \| --- \| \| Anaerotruncus colihominis DSM 17241 \| |
|  | Bacteroides_caccae Bacteroides_ovatus Bacteroides_uniformis Bacteroides_vulgatus | \| Bacteroides fragilis str. 1007-1-F #8 \| \| --- \| \| Bacteroides fragilis str. 3988 T1 \| \| Bacteroides ovatus 3_8_47FAA \| \| Bacteroides ovatus ATCC 8483 \| \| Bacteroides ovatus str. 3725 D1 iv \| \| Bacteroides vulgatus \| \| Bacteroides vulgatus str. 3775 SR(B) \| \| Bacteroides vulgatus str. 3975 RP4 \| \| Bacteroides xylanisolvens \| \| Bacteroides xylanisolvens SD CC 1b \| \| Bacteroides xylanisolvens XB1A \| |
|  | Barnesiella_intestinihominis |  |
|  | Bifidobacterium_adolescentis Bifidobacterium_catenulatum |  |
|  |  | Blautia obeum A2-162 |
|  |  | \| Clostridium botulinum \| \| --- \| \| Clostridium perfringens \| \| Clostridium perfringens WAL-14572 \| \| Clostridium sporogenes \| |
|  | Collinsella_aerofaciens | Collinsella sp. |
|  |  | Coprococcus catus GD/7 |
|  |  | Dorea longicatena |
|  |  | Eggerthella lenta 1_1_60AFAA |
|  |  | Enterococcus faecium 1,230,933 |
|  |  | Escherichia coli O32:H37 str. P4 |
|  |  | \| Eubacterium ramulus ATCC 29099 \| \| --- \| \| Eubacterium rectale \| \| Eubacterium rectale ATCC 33656 \| |
|  | Faecalibacterium_prausnitzii | \| Faecalibacterium prausnitzii L2-6 \| \| --- \| \| Faecalibacterium prausnitzii M21/2 \| |
|  |  | Lactobacillus rogosae |
|  | Lactococcus_lactis Lactococcus_phage |  |
|  | Parabacteroides_merdae | \| Parabacteroides merdae \| \| --- \| \| Parabacteroides merdae CL03T12C32 \| |
|  | Roseburia_hominis | \| Roseburia hominis \| \| --- \| \| Roseburia intestinalis \| \| Roseburia intestinalis L1-82 \| \| Roseburia intestinalis M50/1 \| |
|  | Ruminococcus_lactaris Ruminococcus_torques | \| Ruminococcus bromii L2-63 \| \| --- \| \| Ruminococcus gnavus \| \| Ruminococcus lactaris ATCC 29176 \| |
|  |  | Streptococcus suis GZ1 |
|  | Sutterella_wadsworthensis |  |
| B5 | Akkermansia_muciniphila |  |
|  | Alistipes_putredinis Alistipes_shahii | Alistipes putredinis |
|  | Bacteroides_finegoldii Bacteroides_massiliensis Bacteroides_ovatus Bacteroides_stercoris Bacteroides_uniformis Bacteroides_vulgatus | \| Bacteroides finegoldii CL09T03C10 \| \| --- \| \| Bacteroides ovatus ATCC 8483 \| \| Bacteroides ovatus str. 3725 D1 iv \| \| Bacteroides stercoris \| \| Bacteroides vulgatus \| \| Bacteroides vulgatus PC510 \| \| Bacteroides vulgatus str. 3975 RP4 \| |
|  | Barnesiella_intestinihominis |  |
|  | Bifidobacterium_adolescentis Bifidobacterium_animalis Bifidobacterium_longum | \| Bifidobacterium adolescentis \| \| --- \| \| Bifidobacterium adolescentis L2-32 \| \| Bifidobacterium animalis subsp. lactis AD011 \| \| Bifidobacterium animalis subsp. lactis BB-12 \| \| Bifidobacterium kashiwanohense JCM 15439 = DSM 21854 \| |
|  |  | \| Blautia obeum \| \| --- \| \| Blautia obeum A2-162 \| |
|  |  | \| Clostridium botulinum \| \| --- \| \| Clostridium sporogenes \| |
|  | Collinsella_aerofaciens | Collinsella sp. |
|  | Dialister_invisus | Dialister invisus |
|  |  | Dorea longicatena |
|  |  | Eggerthella lenta DSM 2243 |
|  |  | Enterococcus faecium 1,230,933 |
|  | Eubacterium_eligens | Eubacterium sp. |
|  | Faecalibacterium_prausnitzii | \| Faecalibacterium prausnitzii \| \| --- \| \| Faecalibacterium prausnitzii A2-165 \| \| Faecalibacterium prausnitzii L2-6 \| \| Faecalibacterium prausnitzii M21/2 \| \| Faecalibacterium prausnitzii SL3/3 \| |
|  | Parabacteroides_merdae | \| Parabacteroides merdae \| \| --- \| \| Parabacteroides merdae ATCC 43184 \| \| Parabacteroides merdae CL03T12C32 \| |
|  | Roseburia_intestinalis | \| Roseburia intestinalis M50/1 \| \| --- \| \| Roseburia intestinalis XB6B4 \| |
|  | Ruminococcus_bromii Ruminococcus_callidus Ruminococcus_torques | \| Ruminococcus bromii L2-63 \| \| --- \| \| Ruminococcus lactaris ATCC 29176 \| |
|  | Streptococcus_salivarius |  |
